# Supplementary material for: New Species-Specific Primers for Molecular Diagnosis of Bactrocera minax and Bactrocera tsuneonis (Diptera: Tephritidae) in China Based on DNA Barcodes
Source: Insects. 2019 Dec 12;10(12):447. doi: 10.3390/insects10120447 (PMC6956326; doi:10.3390/insects10120447)
Supplement: Supplementary file 1 [file insects-10-00447-s001.pdf]

Supplemental information

Table S1 Sample information for the 36 *Bactrocera minax* populations and 8 *Bactrocera tsuneonis* populations used in this study

Species	Collection site	Code	Latitude	Longitude	Accession number	
B. minax	Shimen County, Changde City, Hunan Province	SM	29.6536°N	111.0646°E	MK121987<br>MK122016	-
	Hongjiang County, Huaihua City, Hunan Province	HJ	27.2104°N	109.7884°E	MK122052<br>MK122111	-
			27.2208°N	109.7694°E	MK122112<br>MK122144	-
	Jingzhou Miao and Dong Autonomous County，Huaihua City, Hunan Province	JZ	26.6774°N	109.7341°E	MK122145<br>MK122174	-
	Mayang Miao Autonomous County, Huaihua City, Hunan Province	MY	27.8036°N	109.8247°E	MK122175<br>MK122204	-
	Luodian county, Qiannan Buyi and Miao Autonomous Prefecture, Guizhou Province	LD	25.3426°N	106.6638°E	MK124218<br>MK124245	-
	Dongkou County, Shaoyang City, Hunan Province	DK	27.0806°N	110.7209°E	MK122205<br>MK122234	-
	Shaodong County, Shaoyang City, Hunan Province	SD	27.2478°N	111.8964°E	MK122235<br>MK122264	-
			27.2056°N	111.8245°E	MK122265	-

					MK122284
	Xinning County, Shaoyang City,Hunan Province	XN	26.4652°N	110.7256°E	MK122022 - MK122051
			26.5387°N	110.7586°E	MK122285 - MK122298
	Baojing County, Xiangxi Tujia and Miao Autonomous Prefecture, Hunan Province	BJ	28.6154°N	109.4081°E	MK122299 - MK122328
			28.2802°N	109.4581°E	MK122329 - MK122358
	Guzhang County, Xiangxi Tujia and Miao Autonomous Prefecture, Hunan Province	GZ	28.6171°N	109.9508°E	MK122359 - MK122388
	Luxi County, Xiangxi Tujia and Miao Autonomous Prefecture, Hunan Province	LX	28.2341°N	110.0571°E	MK122389 - MK122407
	Yongshun County, Xiangxi Tujia and Miao Autonomous Prefecture, Hunan Province	YS	29.0023°N	109.8069°E	MK122408 - MK122437
			29.0067°N	109.7866°E	MK122438 - MK122467
	Anhua County, Yiyang City, Hunan Province	AH	28.3872°N	111.4763°E	MK122468 - MK122497
	Heshan District, Yiyang City, Hunan Province	HS	28.4391°N	112.4371°E	MK122498 - MK122526
	Sandu County, Qiannan Buyi and Miao Autonomous Prefecture,	QS	25.9372°N	108.0964°E	MK124271 - MK124284

Guizhou Province					
Pingtang County, Qiannan Buyi and Miao Autonomous Prefecture, Guizhou Province	PT	25.8239°N	107.3229°E	MK124246 MK124270	-
Dejiang County, Tongren City, Guizhou Province	DJ	28.3109°N	108.2212°E	MK124295 MK124314	-
		28.1532°N	108.2609°E	MK124315 MK124341	-
		28.3267°N	108.2702°E	MK124342 MK124376	-
Xifeng County, Guiyang City, Guizhou Province	XF	27.2421°N	106.8724°E	MK124150 MK124170	-
Jinping County, Qiandongnan Miao and Dong Autonomous Prefecture, Guizhou Province	JP	26.4544°N	109.2349°E	MK124188 MK124217	-
Duyun City, Qiannan Buyi and Miao Autonomous Prefecture, Guizhou Province	DY	26.2616°N	107.5187°E	MK124171 MK124176	-
Huishui County, Qiandongnan Miao and Dong Autonomous Prefecture, Guizhou Province	QH	26.1213°N	106.6512°E	MK124177 MK124187	-
Daguan County, Zhaotong City, Yunnan Province	DG	27.7491°N	103.8911°E	MK124397 MK124406	-
Yiliang County, Zhaotong City, Yunnan Province	YL	27.6266°N	104.0481°E	MK124407 MK124416	-

	Wanzhou District, Chongqing City	WZ	30.8089°N	108.4087°E	MK124145 MK124149	-
	Dianjiang County, Chongqing City	DI	30.3278°N	107.3352°E	MK124144 MK124140	-
	Pingjiang County, Yueyang City, Hunan Province	PJ	28.7302°N	113.9610°E	MK122527 MK122556	-
	Cili County, Zhangjiajie City, Hunan Province	CL	29.4025°N	111.1753°E	MK122557 MK122586	-
	Changyang Tujia Autonomous County, Yichang City, Hubei Province	CY	30.4739°N	111.2070°E	MK124377 MK124396	-
	Xunyang County, Ankang City, Shaanxi Province	XY	32.8352°N	109.3652°E	MK122587 MK122616	-
	Wengan County, Qiannan Buyi and Miao Autonomous Prefecture, Guizhou Province	WA	27.3139°N	107.4076°E	MK124285 MK124294	-
B.tsuneonis	Pingshan County, Yibin City, Sichuan Province	PS	28.6305°N	103.9361°E	MK124427 MK124435	-
	Jinping County, Qiandongnan Miao and Dong Autonomous Prefecture, Guizhou Province	JP	26.7372°N	109.2410°E	MK124417 MK124418	-
	Libo County, Qiannan Buyi and Miao Autonomous Prefecture, Guizhou Province	LB	25.3978°N	107.9237°E	MK124419 MK124426	-
	Chengbu County, Shaoyang City, Hunan	CB	26.5060°N	110.4192°E	MK122017 MK122021	-

	Province					
	Daguan County, Zhaotong City, Yunan Province	DG	27.7491°N	103.8911°E	MK124464 MK124467	-
	Yongshan County, Zhaotong City, Yunan Province	YZ	28.2016°N	103.6188°E	MK124436 MK124463	-
	Yiliang County, Zhaotong City, Yunan Province	YL	27.6266°N	104.0481°E	MK124468 MK124469	-
	Pingxiang County, Chongzuo City, Guangxi Zhuang Autonomous Region	PX	22.1181°N	106.9205°E	MK163609 MK163615	-

Table S2 Simple information for 658bp standard DNA barcode sequences of *Bactrocera minax, Bactrocera tsuneonis* and other species used in this study.

Genus	Subgenus	Species	Accession number	Collection site
Bactrocera	*Tetradacus*	*B. minax*	JX266430	Guizhou, China
			JX266431	Sichuan, China
			KC346372	China
			KF659877-KF659884	Liupanshui, Guizhou, China
			KF659885-KF659899	Chengdu, Sichuan, China
			KF659900-KF659919	Changde, Hunan, China
			KF659920-KF659929	Yibin, Sichuan, China
		B. tsuneonis	JX266428	Guizhou, China
			JX266429	Sichuan, China
			KF659930-KF659932	Liupanshui, Guizhou, China
			KF659933-KF659947	Yibin, Sichuan, China
	Bactrocera	*B. albistrigata*	JX297522	

B. correcta	JX266410
B. curvipennis	JX266416
B. distincta	KF659788
B. dorsalis	KF659847
B. facialis	DQ116288
B. frauenfeldi	KF659596
B. jarvisi	DQ116260
B. kandiensis	JQ692673
B. kirki	DQ116306
B. latifrons	DQ116310
B. melanotus	JQ692663
B. musae	KF659773
B. neocognata	KF659775
B. nigrotibialis	DQ116340
B. occipitalis	DQ116350
B. paraverbascifoliae	DQ116282
B. psidii	DQ116285
B. redunca	DQ116286
B. rubigina	DQ116290
B. ruiliensis	DQ116298
B. thailandica	DQ116303
B. trilineola	DQ006869
B. trivialis	DQ116312
B. tryoni	DQ116330
B. tuberculata	KF659747
B. umbrosa	DQ116338
B. verbascifoliae	KF659811

		B. wuzhishana	KF659813
		B. zonata	KF659835
	Daculus	*B. oleae*	KF659850
	Gymnodacus	*B. mesomelas*	GQ154150
	Notodacus	*B. xanthodes*	DQ116351
		B. paraxanthodes	DQ116354
Zeugodacus	*Austrodacus*	*Z. cucumis*	DQ116237
	Asiadacus	*B. apiciflava*	KF659854
	Hemigymnodacus	*B. diversus*	KF659858
	Papuodacus	*B. sinensis*	KF659856
	Sinodacus	*B. hochii*	KF659864
	Zeugodacus	*Z. cucurbitae*	JX266418
		Z. scutellatus	JX266426
		Z. tau	JX266422

Table S3 Sample information for primer specificity verification by agarose gel electrophoresis analysis.

Species	Lanes	Collection site
B. minax	1	Xunyang County, Ankang City, Shaanxi Province
	2	Changyang Tujia Autonomous County, Yichang City, Hubei Province
	3	Yongshun County, Xiangxi Tujia and Miao Autonomous Prefecture, Hunan Province
	4	Baojing County, Xiangxi Tujia and Miao Autonomous Prefecture, Hunan Province
	5	Guzhang County, Xiangxi Tujia and Miao Autonomous Prefecture, Hunan Province
	6	Luxi County, Xiangxi Tujia and Miao Autonomous Prefecture, Hunan Province

7	Cili County, Zhangjiajie City, Hunan Province
8	Shimen County, Changde City, Hunan Province
9	Pingjiang County, Yueyang City, Hunan Province
10	Mayang Miao Autonomous County, Huaihua City, Hunan Province
11	Hongjiang County, Huaihua City, Hunan Province
12	Jingzhou Miao and Dong Autonomous County，Huaihua City, Hunan Province
13	Anhua County, Yiyang City, Hunan Province
14	Heshan District, Yiyang City, Hunan Province
15	Dongkou County, Shaoyang City, Hunan Province
16	Shaodong County, Shaoyang City, Hunan Province
17	Xinning County, Shaoyang City,Hunan Province
18	Dejiang County, Tongren City, Guizhou Province
19	Jinping County, Qiandongnan Miao and Dong Autonomous Prefecture, Guizhou Province
20	Wengan County, Qiannan Buyi and Miao Autonomous Prefecture, Guizhou Province
21	Huishui County, Qiandongnan Miao and Dong Autonomous Prefecture, Guizhou Province
22	Sandu County, Qiannan Buyi and Miao Autonomous Prefecture, Guizhou Province
23	Pingtang County, Qiannan Buyi and Miao Autonomous Prefecture, Guizhou Province
24	Luodian county, Qiannan Buyi and Miao Autonomous Prefecture, Guizhou Province
25	Xifeng County, Guiyang City, Guizhou Province
26	Daguan County, Zhaotong City, Yunnan Province
27	Dianjiang County, Chongqing City
28	Yiliang County, Zhaotong City, Yunnan Province

	29	Wanzhou District, Chongqing City
B. tsuneonis	30	Pingshan County, Yibin City, Sichuan Province
	31	Libo County, Qiannan Buyi and Miao Autonomous Prefecture, Guizhou Province
	32	Jinping County, Qiandongnan Miao and Dong Autonomous Prefecture, Guizhou Province
	33	Yongshan County, Zhaotong City, Yunan Province
	34	Pingxiang County, Chongzuo City, Guangxi Zhuang Autonomous Region
	35	Pingshan County, Yibin City, Sichuan Province
	36	Yiliang County, Zhaotong City, Yunan Province
	37	Daguan County, Zhaotong City, Yunan Province
	38	Chengbu County, Shaoyang City, Hunan Province
B. correcta	39	Yuanjiang Hani, Yi and Dai Autonomous County, Yuxi City, Yunnan Province
B. dorsalis	40	Huadu District, Guangzhou City, Guangdong Province
B. latifrons	41	Mengla County, Xishuangbanna Dai Autonomous Prefecture, Yunnan Province
B. tryoni	42	Feed in IAEA
B. zonata	43	Feed in IAEA
Zeugodacus cucurbitae	44	Mengla County, Xishuangbanna Dai Autonomous Prefecture, Yunnan Province
Z. scutellatus	45	Mengla County, Xishuangbanna Dai Autonomous Prefecture, Yunnan Province
Z. tau	46	Mengla County, Xishuangbanna Dai Autonomous Prefecture, Yunnan Province

Table S4 Haplotypes of *Bactrocera minax* and *Bactrocera tsuneonis* included in this study.

Species	Haplotypes number	Sequences
B. tsuneonis	Hap-1	MK163609, MK163610, MK163612,

		MK163614
	Hap-2	MK163611, MK163613, MK163615, MK124419 - MK124422, MK124424, MK124425, MK124429, MK124430, MK124432, MK124433, MK124437, MK124438, MK124440, MK124442, MK124444, MK124445, MK124448, MK124449, MK124451, MK124452, MK124457, MK124458, MK124462, MK124468
	Hap-3	MK124417, MK124418, MK122019
	Hap-4	MK124423, MK124426, MK124441, MK124447, MK124453, MK124454, MK124456
	Hap-5	MK122017
	Hap-6	MK122018, MK122021
	Hap-7	MK122022
	Hap-8	MK124427, MK124431, MK124435
	Hap-9	MK124434
	Hap-10	MK124436
	Hap-11	MK124439
	Hap-12	MK124443, MK124446, MK124450, MK124455, MK124459 - MK124461, MK124464 - MK124467, MK124469
B. minax	Hap-1	MK122587
	Hap-2	MK122588, MK122590, MK122598, MK122602, MK122603, MK122604, MK122606, MK122609, MK122613, MK122616, MK122408 - MK122413, MK122418, MK122431, MK122433, MK124378 - MK124389, MK124391, MK124393 - MK124395, MK122527 - MK122556
	Hap-3	MK122589, MK122608, MK121993, MK121995, MK121997 - MK121999,

		MK122001 - MK122011, MK124140, MK124141, MK124144, MK124145, MK124147, MK124153, MK124154, MK124166 - MK124170, MK122052 - MK122057, MK122117 - MK122124, MK122130 - MK122134, MK122175, MK122176, MK122179, MK122183, MK124177, MK124180, MK124194 - MK124198, MK122210 - MK122216, MK122222 - MK122226, MK122229, MK122235, MK122237, MK122248 - MK122264, MK122265, MK122267 - MK122276, MK122278 - MK122283, MK122030, MK122032 - MK122051, MK122292, MK122298, MK122299 - MK122303, MK122305 - MK122309, MK122312, MK122318, MK122324, MK122330, MK122336, MK122359 - MK122388, MK122401, MK122402, MK122414 - MK122417, MK122419 - MK122423, MK122425 - MK122430, MK122432, MK122434 - MK122436, MK122438 - MK122442, MK122448, MK122449, MK122451, MK122452, MK122458, MK122459, MK122461 - MK122464, MK122466, MK122467, MK124396, MK122469, MK122472, MK122474, MK122475, MK122478, MK122479, MK122481 - MK122483, MK122485, MK122486, MK122489 - MK122491, MK122503 - MK122525 , MK124407, MK124409, MK124412, MK124414, MK122557 - MK122561, MK122563 - MK122586
	Hap-4	MK122591, MK122611, MK122612
	Hap-5	MK122592
	Hap-6	MK122593, MK122595, MK122597, MK122599, MK122605, MK122607

Hap-7	MK122594, MK122596, MK122610, MK122615, MK122319 - MK122323, MK122325, MK122326, MK124392, MK124398, MK124399
Hap-8	MK122600, MK122614, MK121987 - MK121991, MK122000, MK124146, MK124148, MK124346 - MK124349, MK122344 - MK122348, MK124377, MK124403
Hap-9	MK122601, MK124142, MK124143, MK122277, MK122284, MK122498 - MK122502, MK124397, MK124408, MK124410, MK124411, MK124413, MK124415
Hap-10	MK121992, MK121994, MK121996, MK122008 - MK122012, MK124149, MK122057 - MK122071, MK122082 - MK122106, MK122125 - MK122129, MK122135 - MK122144, MK122145 - MK122154, MK122165 - MK122174, MK122180 - MK122182, MK122186, MK122189 - MK122197, MK122205 - MK122209, MK122217 - MK122221, MK122227, MK122228, MK122230 - MK122234, MK122238 - MK122243, MK122245, MK122391 - MK122395, MK122398 - MK122400, MK122403 - MK122405, MK122407
Hap-11	MK124150 - MK124152, MK124161, MK122062 - MK122066, MK122072 - MK122076, MK122185 - MK122187, MK122189, MK122190, MK122201 - MK122204, MK124188 - MK124193, MK124205 - MK124217, MK124218 - MK124226, MK124257, MK124259 - MK124262, MK124265, MK124267, MK124192
Hap-12	MK124155 - MK124160, MK124172, MK124175, MK124176, MK124181,

		MK124182, MK124184, MK124187, MK124227 - MK124245, MK124285 - MK124294, MK124295 - MK124314, MK124315 - MK124318, MK124320 - MK124328, MK124335, MK124337 - MK124341, MK124342 - MK124376, MK124401, MK124405
	Hap-13	MK124162 - MK124165
	Hap-14	MK124171
	Hap-15	MK124173
	Hap-16	MK124174
	Hap-17	MK122077 - MK122081, MK122107 - MK122111, MK122112 - MK122116, MK122160 - MK122164 , MK124199 - MK124204 , MK122022 - MK122029 , MK122031, MK122291
	Hap-18	MK122177, MK122178
	Hap-19	MK122200
	Hap-20	MK124178, MK124179, MK124247, MK124248, MK124271 - MK124284
	Hap-21	MK124183, MK124185, MK124186
	Hap-22	MK124246, MK124250, MK124258, MK124263, MK124266, MK124268 - MK124270
	Hap-23	MK124249, MK124264
	Hap-24	MK124251, MK124253, MK124255, MK124256
	Hap-25	MK124252, MK124254
	Hap-26	MK122236, MK122247, MK122305
	Hap-27	MK122244
	Hap-28	MK122246

Hap-29	MK122266
Hap-30	MK122285, MK122288
Hap-31	MK122293, MK122294
Hap-32	MK122295, MK122297
Hap-33	MK122296
Hap-34	MK122286
Hap-35	MK122287, MK122289, MK122290
Hap-36	MK124329 - MK124334, MK124336
Hap-37	MK122310 - MK122318, MK122443 - MK122447, MK122465
Hap-38	MK122317
Hap-39	MK122329, MK122331, MK122333 - MK122335, MK122337 - MK122350, MK122352 - MK122358
Hap-40	MK122332, MK122351
Hap-41	MK122389, MK122390, MK122396, MK122397, MK122406
Hap-42	MK122424
Hap-43	MK122450, MK122460
Hap-44	MK124390
Hap-45	MK122468, MK122470, MK122471, MK122473, MK122476, MK122477, MK122480, MK122487, MK122488, MK122493
Hap-46	MK122484, MK122497, MK122489
Hap-47	MK122492, MK122494 - MK122496
Hap-48	MK124400, MK124406
Hap-49	MK124402, MK124416

	Hap-50	MK124404
	Hap-51	MK122562

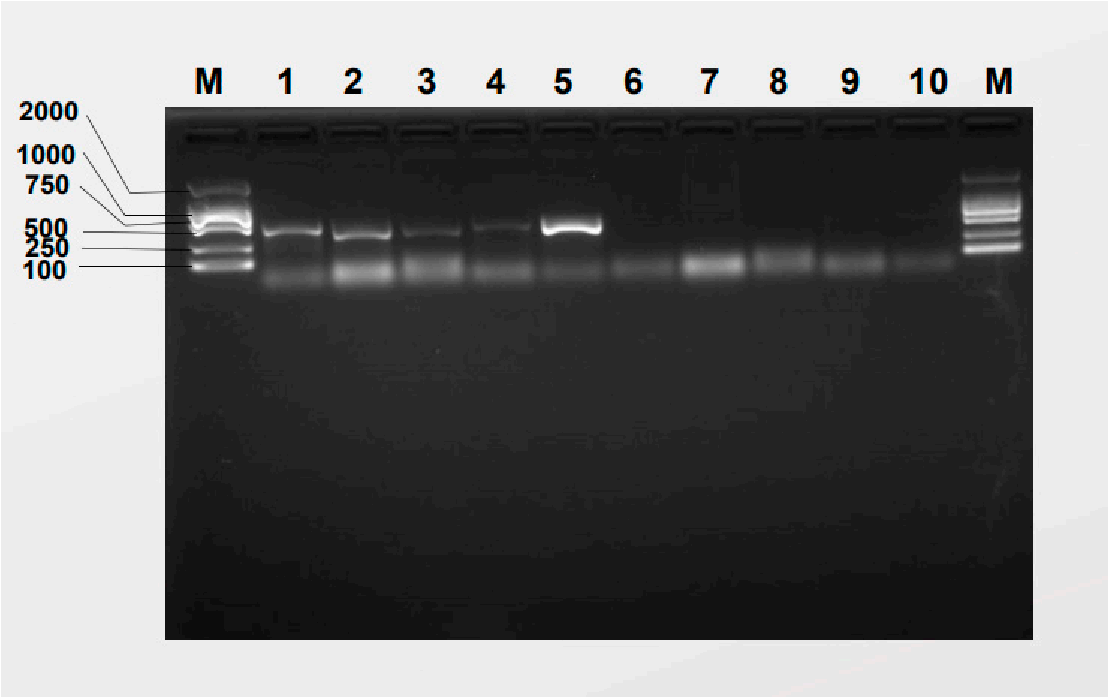

Figure S1 Specificity of the Bm-F/Bm-R *B. minax*-specific primer pair and the Bt-F/Bt-R *B. tsuneonis*-specific primer pair, Lanes 1-5: *B. minax*-specific primer pair DNA amplification for 5 *B. minax* sample from Xunyang County, Ankang City, Shaanxi Province; Lanes 6-10: *B. tsuneonis*-specific primer pair DNA amplification of 5 *B. minax* sample from Xunyang County, Ankang City, Shaanxi Province; Lane M: D2000.

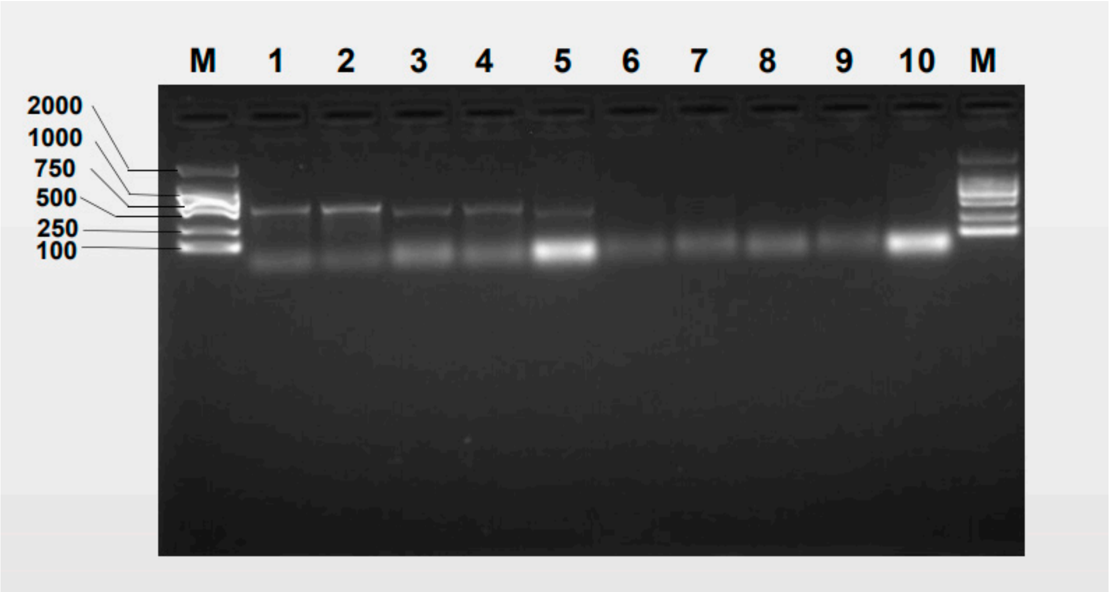

Figure S2 Specificity of the Bm-F/Bm-R *B. minax*-specific primer pair and the Bt-F/Bt-R *B. tsuneonis*-specific primer pair, Lanes 1-5: *B. minax*-specific primer pair DNA amplification for 5 *B. minax* sample from Changyang Tujia Autonomous County, Yichang City, Hubei Province; Lanes 6-10: *B. tsuneonis*-specific primer pair DNA amplification for 5 *B. minax* sample from Changyang Tujia Autonomous County, Yichang City, Hubei Province; Lane M: D2000.

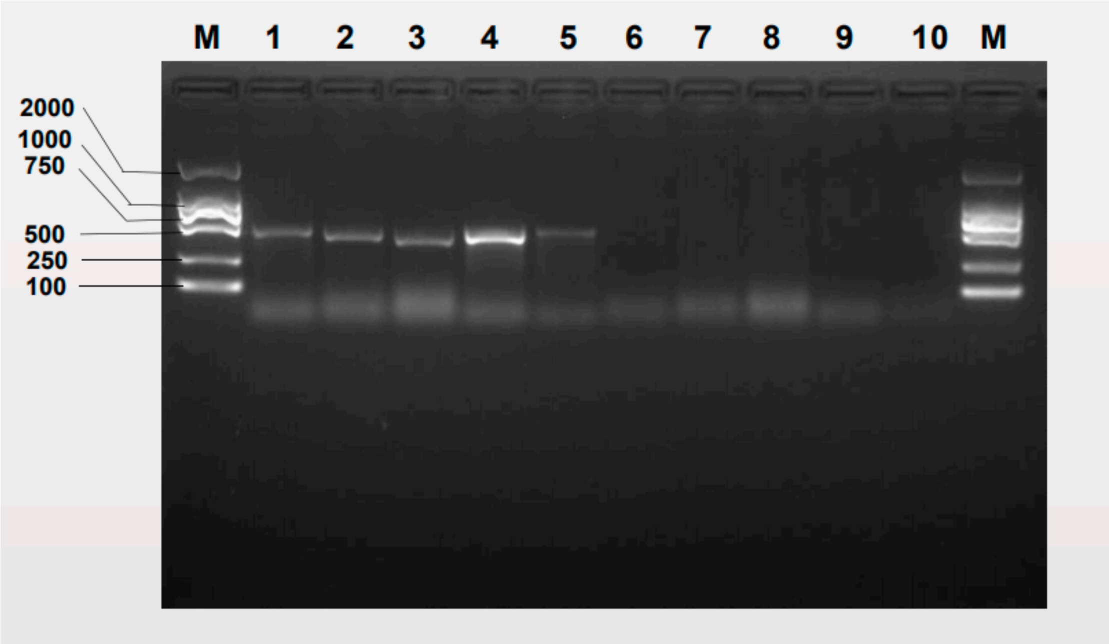

Figure S3 Specificity of the Bm-F/Bm-R *B. minax*-specific primer pair and the Bt-F/Bt-R *B. tsuneonis*-specific primer pair, Lanes 1-5: *B. minax*-specific primer pair DNA amplification for 5 *B. minax* sample from Yongshun County, Xiangxi Tujia and Miao Autonomous Prefecture, Hunan Province; Lanes 6-10: *B. tsuneonis*-specific primer pair DNA amplification for 5 *B. minax* sample

from Yongshun County, Xiangxi Tujia and Miao Autonomous Prefecture, Hunan Province; Lane M: D2000.

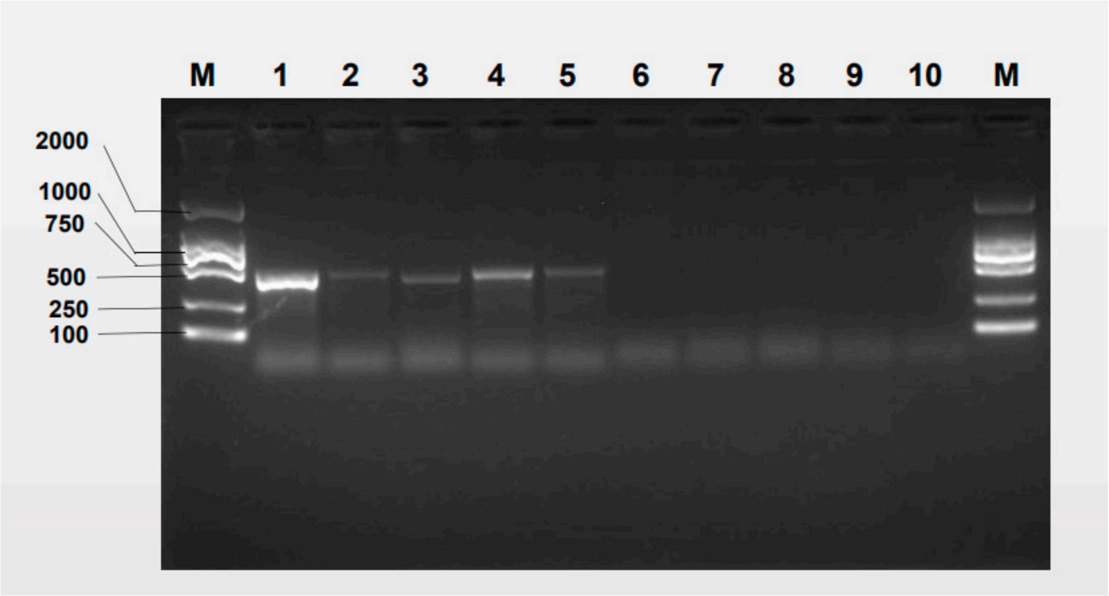

Figure S4 Specificity of the Bm-F/Bm-R *B. minax*-specific primer pair and the Bt-F/Bt-R *B. tsuneonis*-specific primer pair, Lanes 1-5: *B. minax*-specific primer pair DNA amplification for 5 *B. minax* sample from Baojing County, Xiangxi Tujia and Miao Autonomous Prefecture, Hunan Province; Lanes 6-10: *B. tsuneonis*-specific primer pair DNA amplification for 5 *B. minax* sample from Baojing County, Xiangxi Tujia and Miao Autonomous Prefecture, Hunan Province; Lane M: D2000.

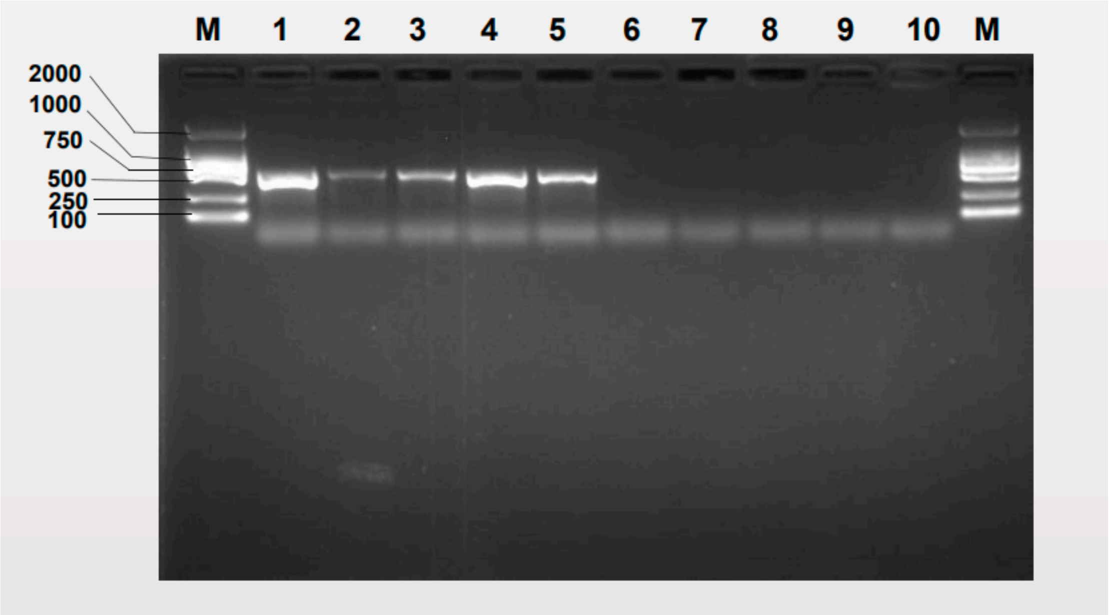

Figure S5 Specificity of the Bm-F/Bm-R *B. minax*-specific primer pair and the Bt-F/Bt-R *B. tsuneonis*-specific primer pair, Lanes 1-5: *B. minax*-specific primer pair DNA amplification for 5 *B. minax* sample from Guzhang County, Xiangxi Tujia and Miao Autonomous Prefecture, Hunan Province; Lanes 6-10: *B. tsuneonis*-specific primer pair DNA amplification for 5 *B. minax* sample from Guzhang County, Xiangxi Tujia and Miao Autonomous Prefecture, Hunan Province; Lane M: D2000.

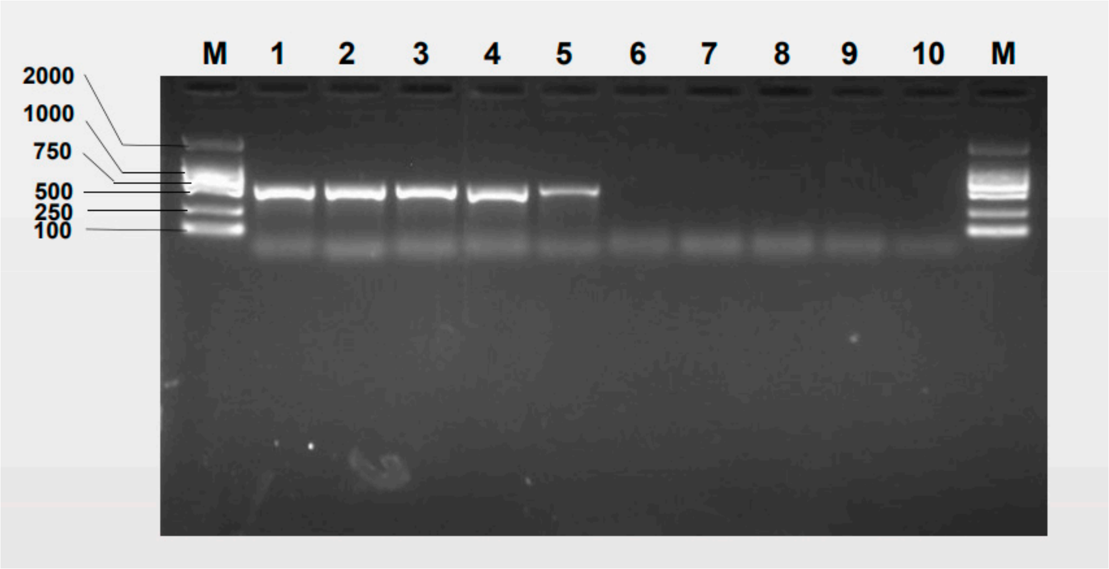

Figure S6 Specificity of the Bm-F/Bm-R *B. minax*-specific primer pair and the Bt-F/Bt-R *B. tsuneonis*-specific primer pair, Lanes 1-5: *B. minax*-specific primer pair DNA amplification for 5 *B. minax* sample from Luxi County, Xiangxi Tujia and Miao Autonomous Prefecture, Hunan Province; Lanes 6-10: *B. tsuneonis*-specific primer pair DNA amplification for 5 *B. minax* sample from Luxi County, Xiangxi Tujia and Miao Autonomous Prefecture, Hunan Province; Lane M: D2000.

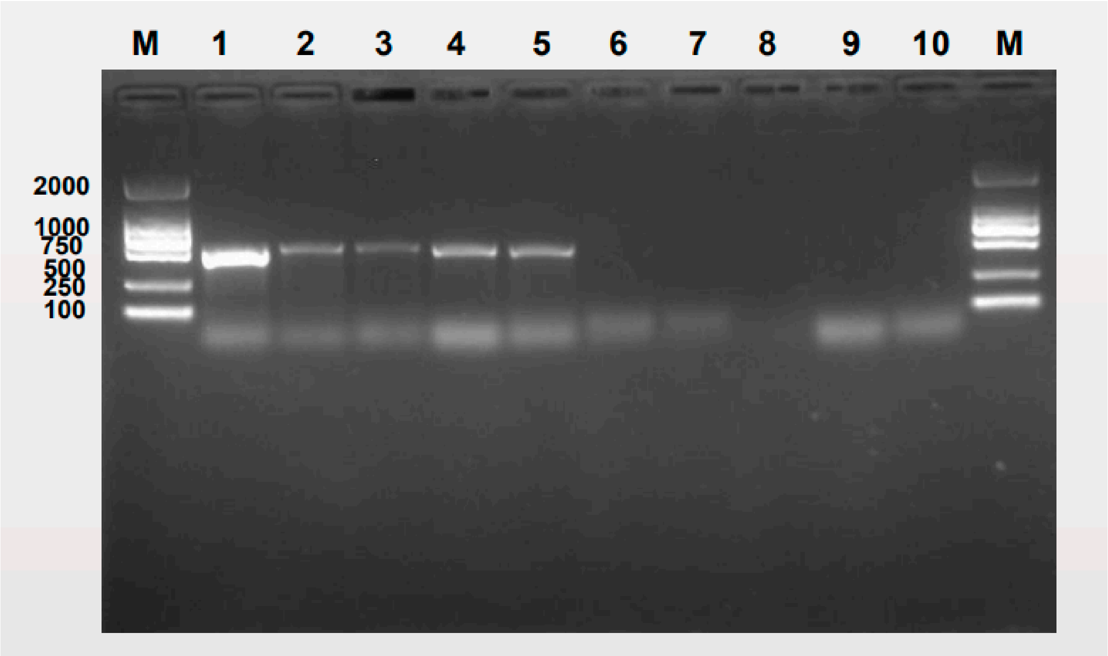

Figure S7 Specificity of the Bm-F/Bm-R *B. minax*-specific primer pair and the Bt-F/Bt-R *B. tsuneonis*-specific primer pair, Lanes 1-5: *B. minax*-specific primer pair DNA amplification for 5 *B. minax* sample from Cili County, Zhangjiajie City, Hunan Province; Lanes 6-10: *B. tsuneonis*-specific primer pair DNA amplification for 5 *B. minax* sample from Cili County, Zhangjiajie City, Hunan Province; Lane M: D2000.

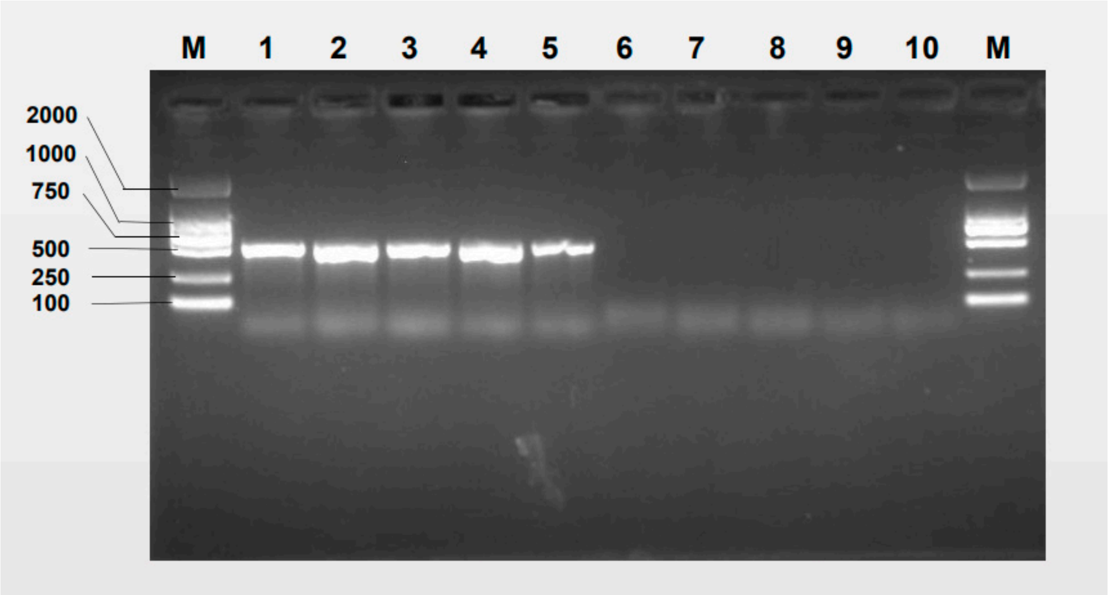

Figure S8 Specificity of the Bm-F/Bm-R *B. minax*-specific primer pair and the Bt-F/Bt-R *B. tsuneonis*-specific primer pair, Lanes 1-5: *B. minax*-specific primer pair DNA amplification for 5

B. minax sample from Shimen County, Changde City, Hunan Province; Lanes 6-10: *B. tsuneonis*-specific primer pair DNA amplification for 5 *B. minax* sample from Shimen County, Changde City, Hunan Province; Lane M: D2000.

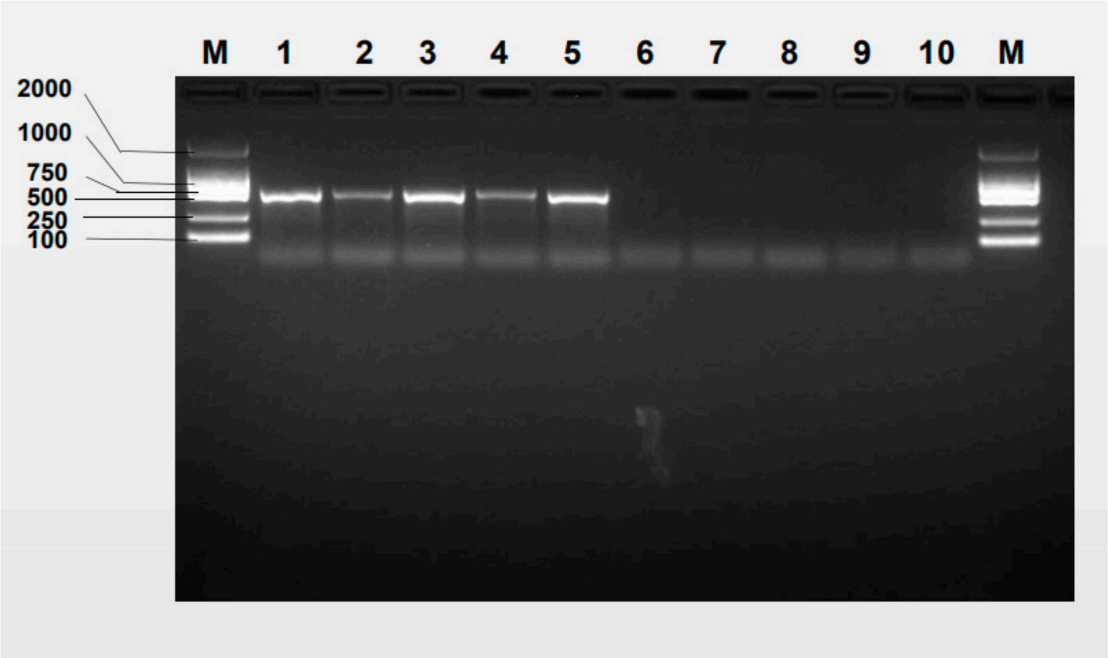

Figure S9 Specificity of the Bm-F/Bm-R *B. minax*-specific primer pair and the Bt-F/Bt-R *B. tsuneonis*-specific primer pair, Lanes 1-5: *B. minax*-specific primer pair DNA amplification for 5 *B. minax* sample from Pingjiang County, Yueyang City, Hunan Province; Lanes 6-10: *B. tsuneonis*-specific primer pair DNA amplification for 5 *B. minax* sample from Pingjiang County, Yueyang City, Hunan Province; Lane M: D2000.

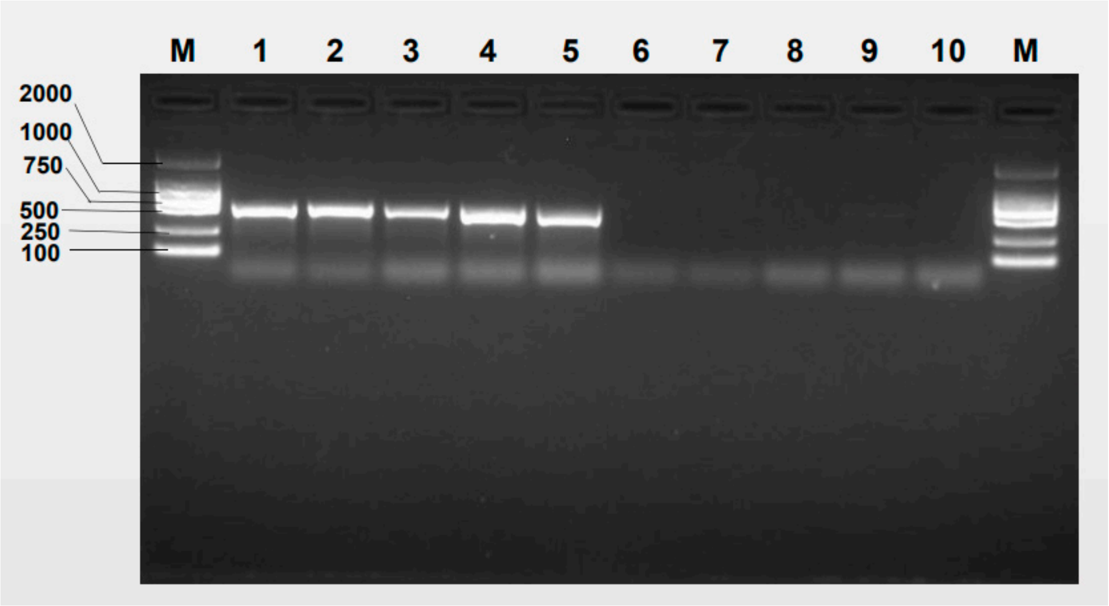

Figure S10 Specificity of the Bm-F/Bm-R *B. minax*-specific primer pair and the Bt-F/Bt-R *B. tsuneonis*-specific primer pair, Lanes 1-5: *B. minax*-specific primer pair DNA amplification for 5 *B. minax* sample from Mayang Miao Autonomous County, Huaihua City, Hunan Province; Lanes 6-10: *B. tsuneonis*-specific primer pair DNA amplification for 5 *B. minax* sample from Mayang Miao Autonomous County, Huaihua City, Hunan Province; Lane M: D2000.

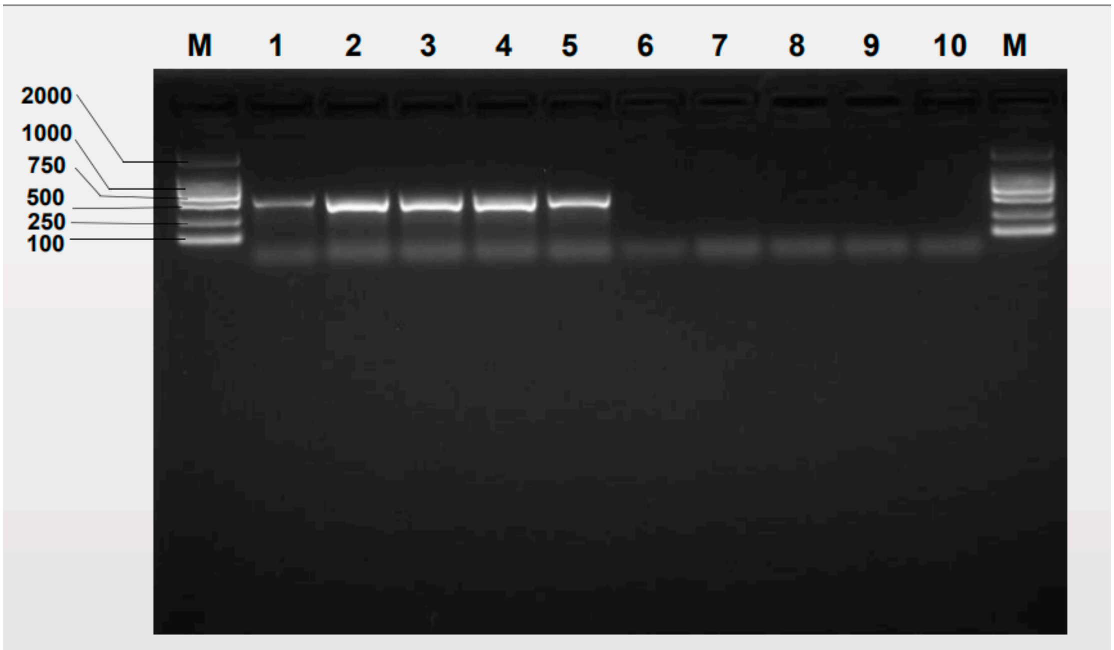

Figure S11 Specificity of the Bm-F/Bm-R *B. minax*-specific primer pair and the Bt-F/Bt-R *B. tsuneonis*-specific primer pair, Lanes 1-5: *B. minax*-specific primer pair DNA amplification for 5 *B. minax* sample from Hongjiang County, Huaihua City, Hunan Province; Lanes 6-10: *B. tsuneonis*-specific primer pair DNA amplification for 5 *B. minax* sample from Hongjiang County,

Huaihua City, Hunan Province; Lane M: D2000.

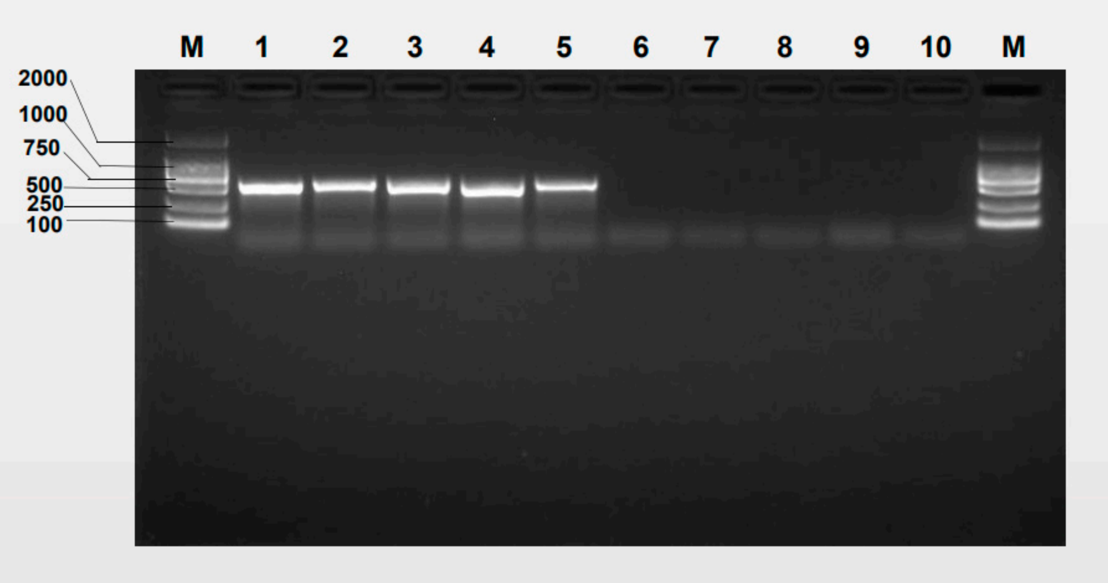

Figure S12 Specificity of the Bm-F/Bm-R *B. minax*-specific primer pair and the Bt-F/Bt-R *B. tsuneonis*-specific primer pair, Lanes 1-5: *B. minax*-specific primer pair DNA amplification for 5 *B. minax* sample from Jingzhou Miao and Dong Autonomous County，Huaihua City, Hunan Province; Lanes 6-10: *B. tsuneonis*-specific primer pair DNA amplification for 5 *B. minax* sample from Jingzhou Miao and Dong Autonomous County，Huaihua City, Hunan Province; Lane M: D2000.

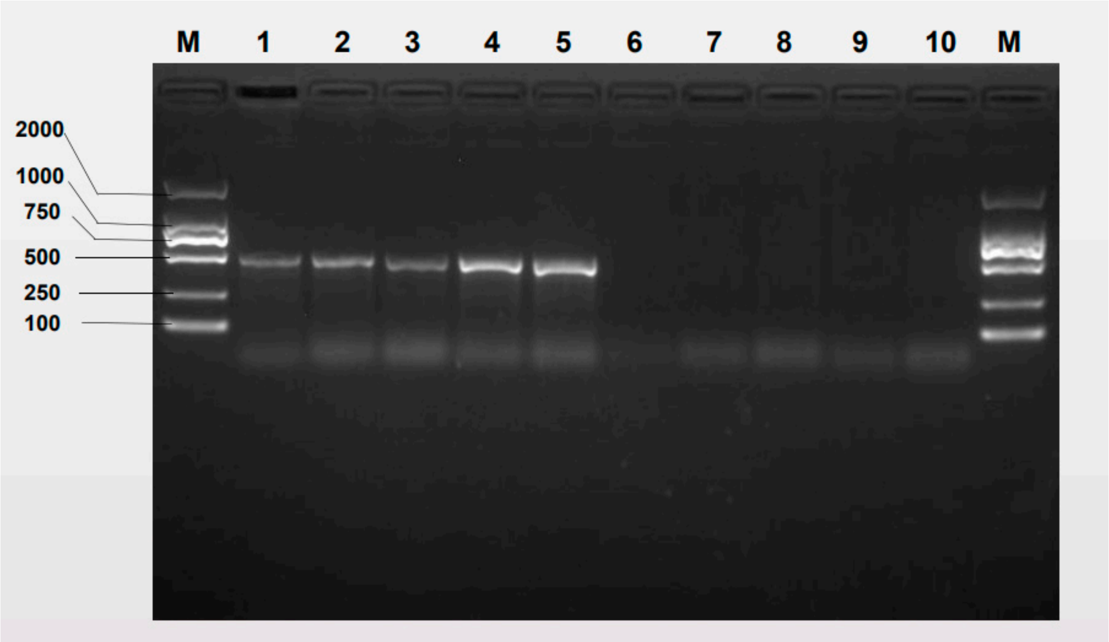

Figure S13 Specificity of the Bm-F/Bm-R *B. minax*-specific primer pair and the Bt-F/Bt-R *B. tsuneonis*-specific primer pair, Lanes 1-5: *B. minax*-specific primer pair DNA amplification for 5 *B. minax* sample from Anhua County, Yiyang City, Hunan Province; Lanes 6-10: *B. tsuneonis*-specific primer pair DNA amplification for 5 *B. minax* sample from Anhua County, Yiyang City, Hunan Province; Lane M: D2000.

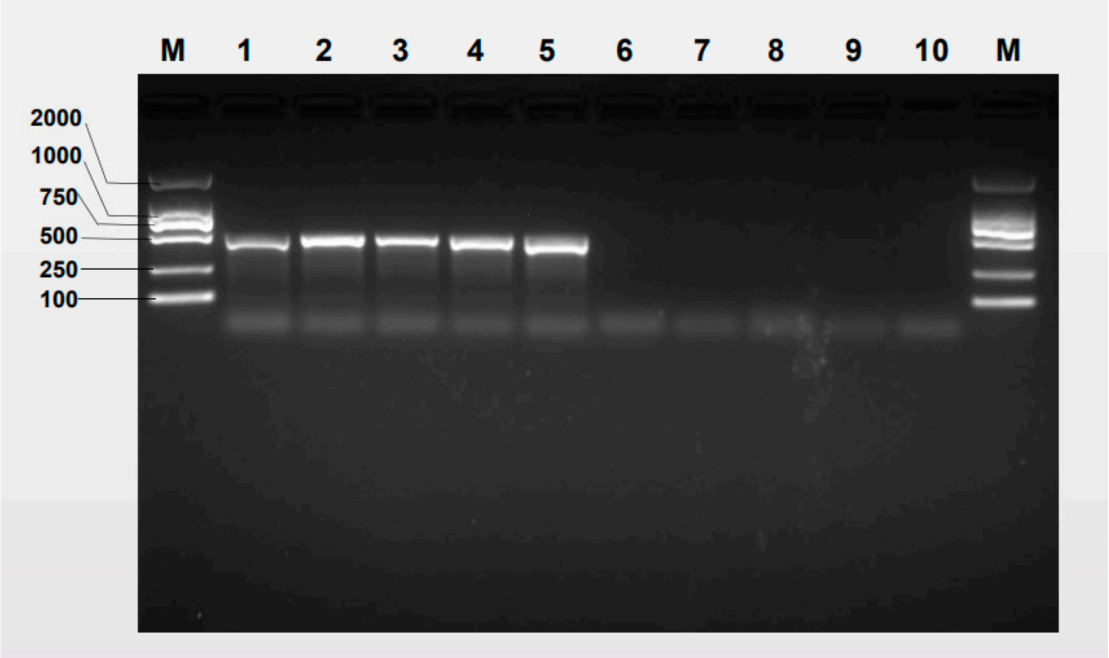

Figure S14 Specificity of the Bm-F/Bm-R *B. minax*-specific primer pair and the Bt-F/Bt-R *B. tsuneonis*-specific primer pair, Lanes 1-5: *B. minax*-specific primer pair DNA amplification for 5 *B. minax* sample from Heshan District, Yiyang City, Hunan Province; Lanes 6-10: *B.*

tsuneonis-specific primer pair DNA amplification for 5 *B. minax* sample from Heshan District, Yiyang City, Hunan Province; Lane M: D2000.

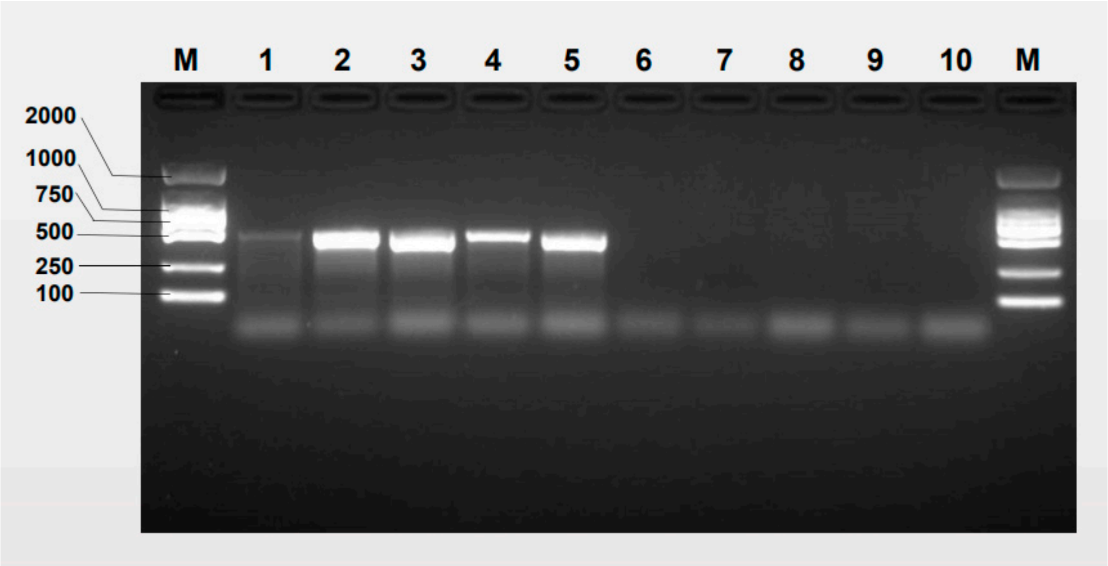

Figure S15 Specificity of the Bm-F/Bm-R *B. minax*-specific primer pair and the Bt-F/Bt-R *B. tsuneonis*-specific primer pair, Lanes 1-5: *B. minax*-specific primer pair DNA amplification for 5 *B. minax* sample from Dongkou County, Shaoyang City, Hunan Province; Lanes 6-10: *B. tsuneonis*-specific primer pair DNA amplification for 5 *B. minax* sample from Dongkou County, Shaoyang City, Hunan Province; Lane M: D2000.

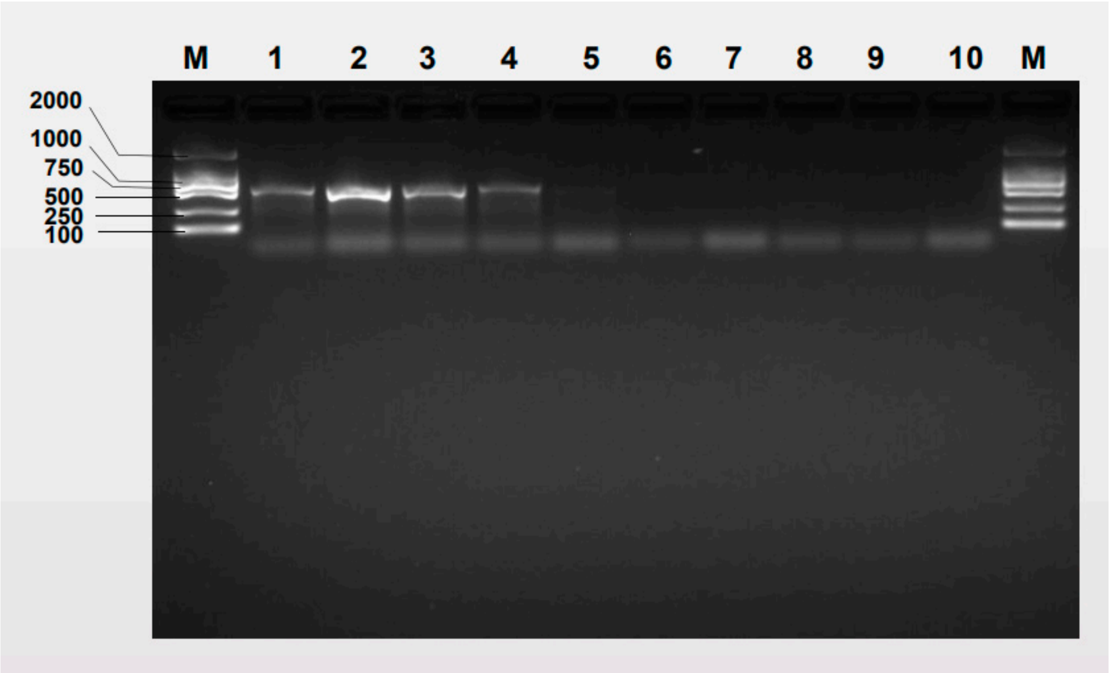

Figure S16 Specificity of the Bm-F/Bm-R *B. minax*-specific primer pair and the Bt-F/Bt-R *B. tsuneonis*-specific primer pair, Lanes 1-5: *B. minax*-specific primer pair DNA amplification for 5 *B. minax* sample from Shaodong County, Shaoyang City, Hunan Province; Lanes 6-10: *B. tsuneonis*-specific primer pair DNA amplification for 5 *B. minax* sample from Shaodong County, Shaoyang City, Hunan Province; Lane M: D2000.

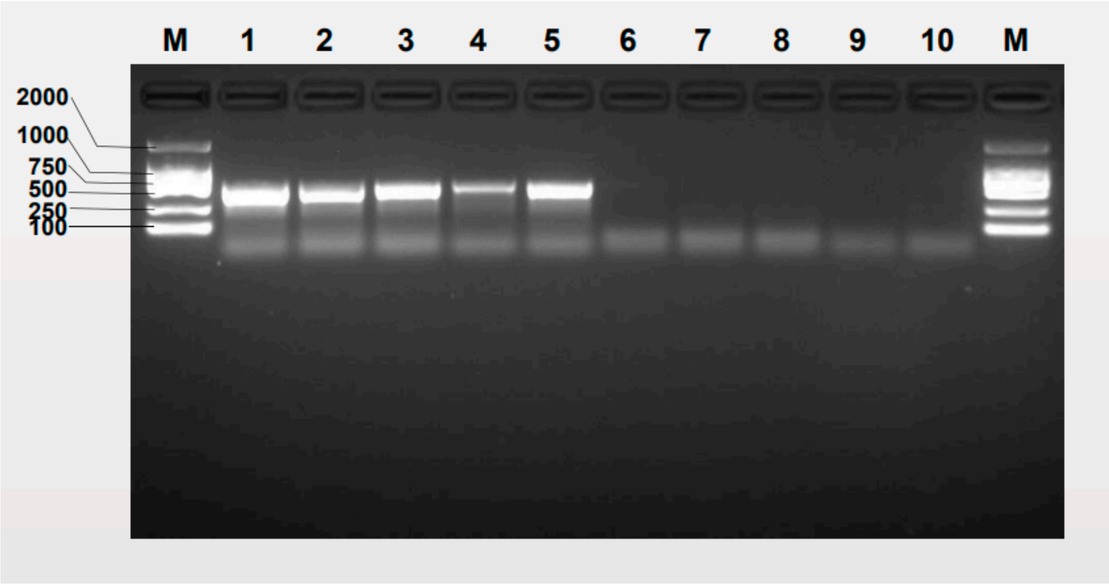

Figure S17 Specificity of the Bm-F/Bm-R *B. minax*-specific primer pair and the Bt-F/Bt-R *B. tsuneonis*-specific primer pair, Lanes 1-5: *B. minax*-specific primer pair DNA amplification for 5 *B. minax* sample from Xinning County, Shaoyang City,Hunan Province; Lanes 6-10: *B.*

tsuneonis-specific primer pair DNA amplification for 5 *B. minax* sample from Xinning County, Shaoyang City,Hunan Province; Lane M: D2000.

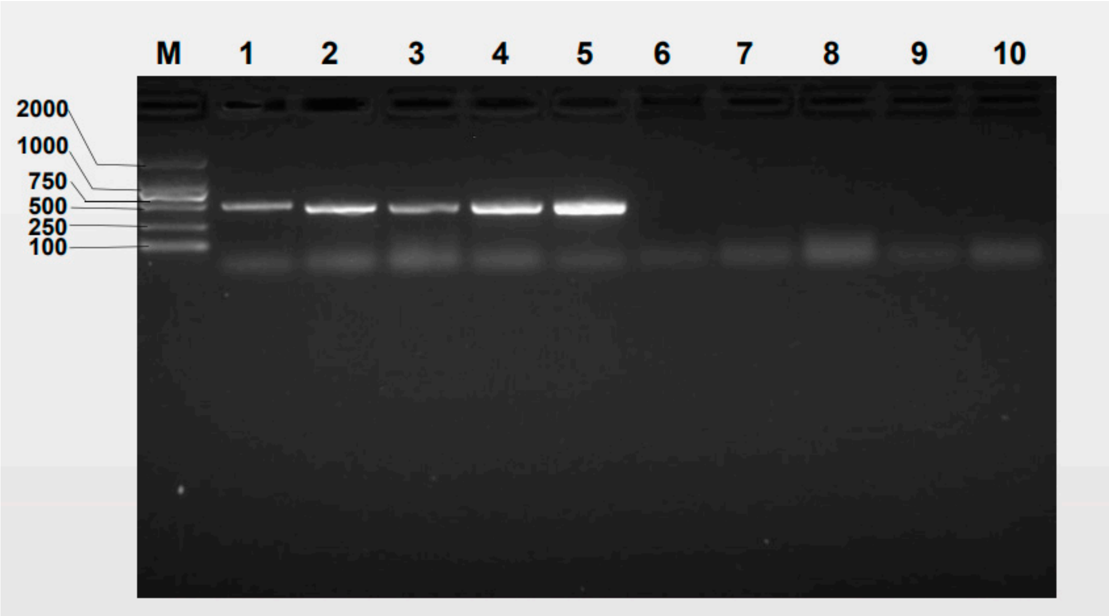

Figure S18 Specificity of the Bm-F/Bm-R *B. minax*-specific primer pair and the Bt-F/Bt-R *B. tsuneonis*-specific primer pair, Lanes 1-5: *B. minax*-specific primer pair DNA amplification for 5 *B. minax* sample from Dejiang County, Tongren City, Guizhou Province; Lanes 6-10: *B. tsuneonis*-specific primer pair DNA amplification for 5 *B. minax* sample from Dejiang County, Tongren City, Guizhou Province; Lane M: D2000.

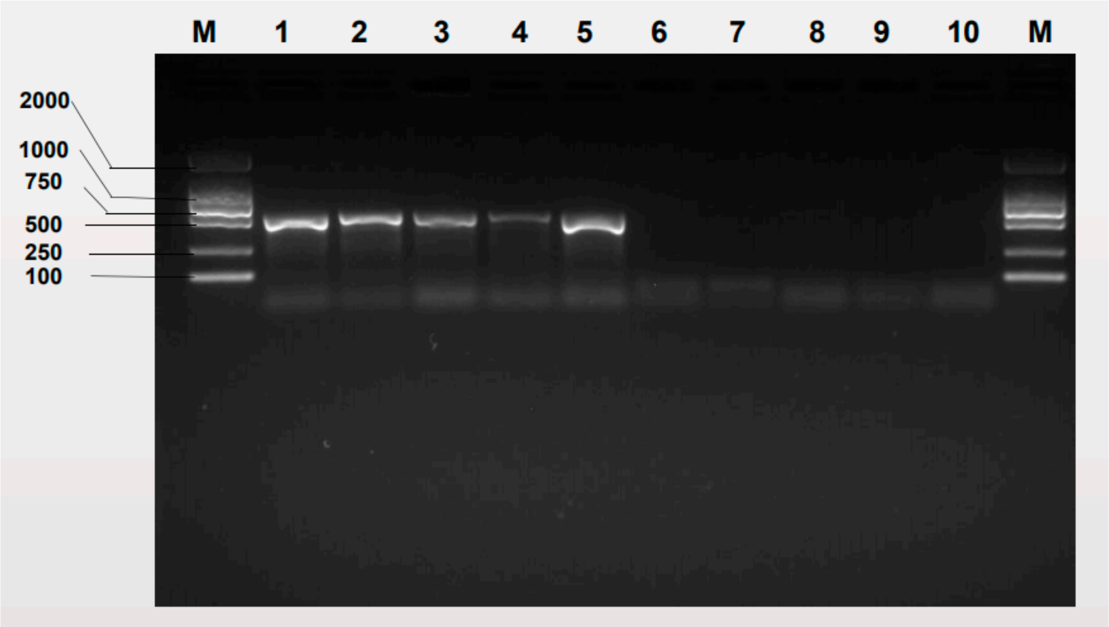

Figure S19 Specificity of the Bm-F/Bm-R *B. minax*-specific primer pair and the Bt-F/Bt-R *B. tsuneonis*-specific primer pair, Lanes 1-5: *B. minax*-specific primer pair DNA amplification for 5 *B. minax* sample from Jinping County, Qiandongnan Miao and Dong Autonomous Prefecture, Guizhou Province; Lanes 6-10: *B. tsuneonis*-specific primer pair DNA amplification for 5 *B. minax* sample from Jinping County, Qiandongnan Miao and Dong Autonomous Prefecture, Guizhou Province; Lane M: D2000.

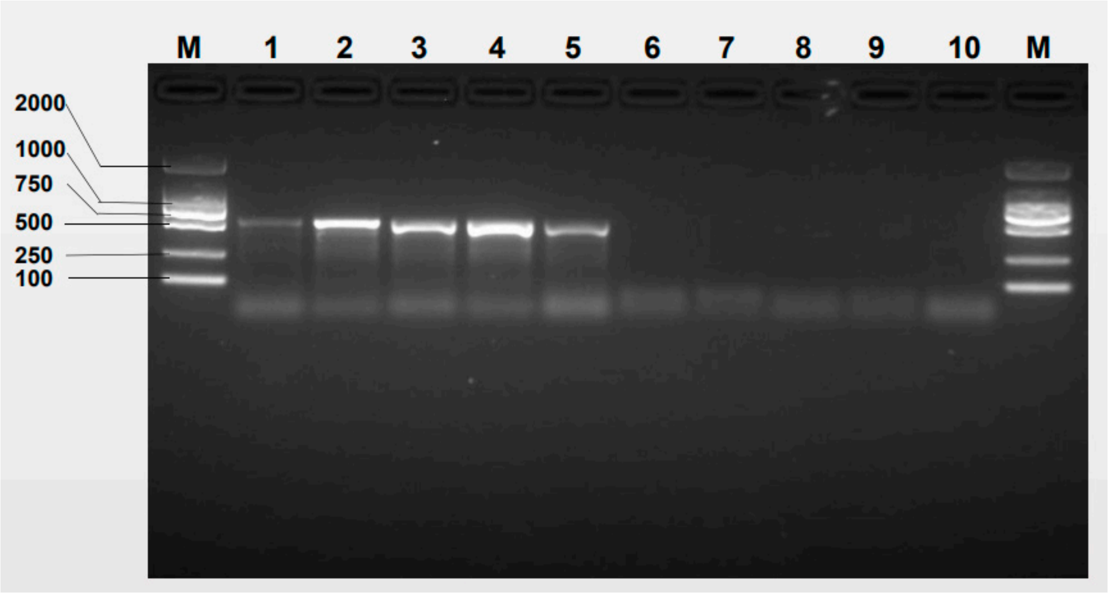

Figure S20 Specificity of the Bm-F/Bm-R *B. minax*-specific primer pair and the Bt-F/Bt-R *B. tsuneonis*-specific primer pair, Lanes 1-5: *B. minax*-specific primer pair DNA amplification for 5 *B. minax* sample from Wengan County, Qiannan Buyi and Miao Autonomous Prefecture, Guizhou Province; Lanes 6-10: *B. tsuneonis*-specific primer pair DNA amplification for 5 *B. minax* sample

from Wengan County, Qiannan Buyi and Miao Autonomous Prefecture, Guizhou Province; Lane M: D2000.

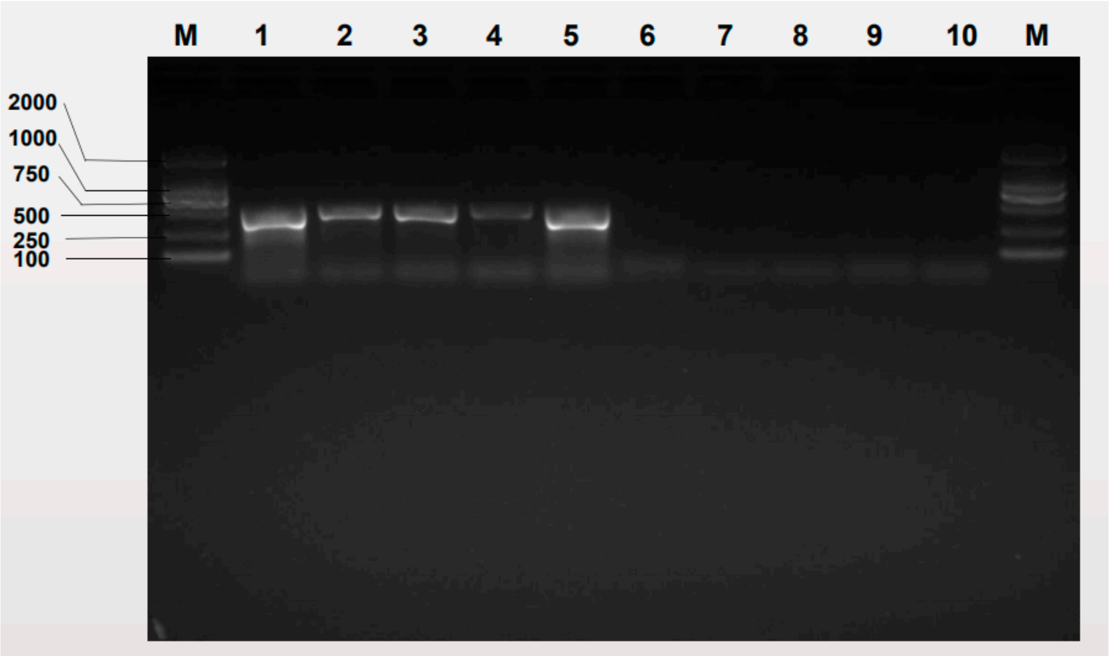

Figure S21 Specificity of the Bm-F/Bm-R *B. minax*-specific primer pair and the Bt-F/Bt-R *B. tsuneonis*-specific primer pair, Lanes 1-5: *B. minax*-specific primer pair DNA amplification for 5 *B. minax* sample from Huishui County, Qiandongnan Miao and Dong Autonomous Prefecture, Guizhou Province; Lanes 6-10: *B. tsuneonis*-specific primer pair DNA amplification for 5 *B. minax* sample from Huishui County, Qiandongnan Miao and Dong Autonomous Prefecture, Guizhou Province; Lane M: D2000.

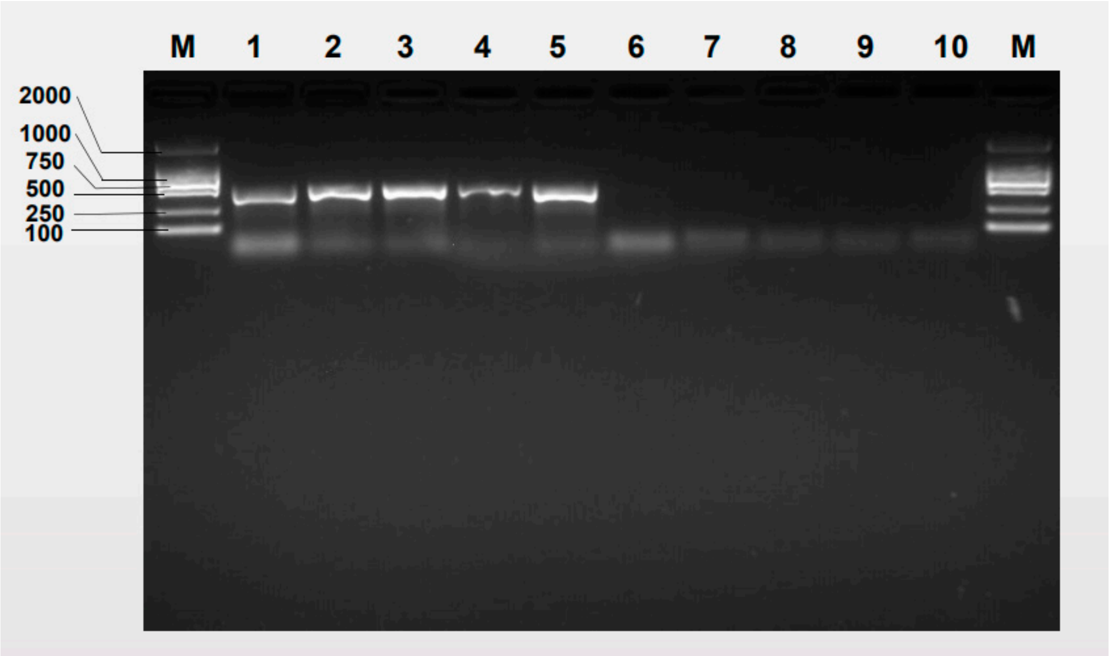

Figure S22 Specificity of the Bm-F/Bm-R *B. minax*-specific primer pair and the Bt-F/Bt-R *B. tsuneonis*-specific primer pair, Lanes 1-5: *B. minax*-specific primer pair DNA amplification for 5 *B. minax* sample from Sandu County, Qiannan Buyi and Miao Autonomous Prefecture, Guizhou Province; Lanes 6-10: *B. tsuneonis*-specific primer pair DNA amplification for 5 *B. minax* sample from Sandu County, Qiannan Buyi and Miao Autonomous Prefecture, Guizhou Province; Lane M: D2000.

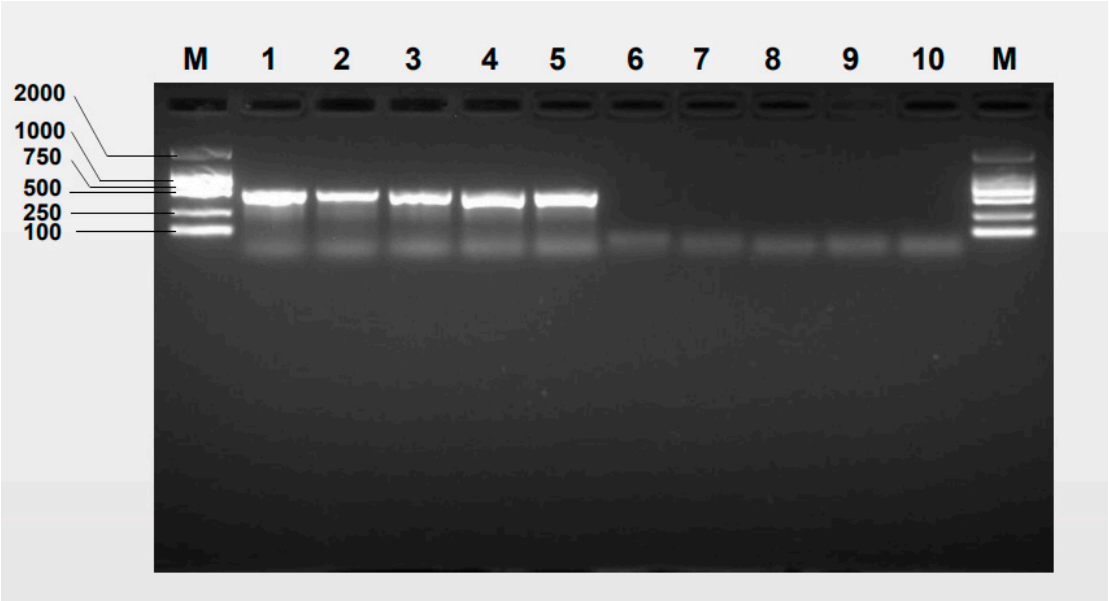

Figure S23 Specificity of the Bm-F/Bm-R *B. minax*-specific primer pair and the Bt-F/Bt-R *B. tsuneonis*-specific primer pair, Lanes 1-5: *B. minax*-specific primer pair DNA amplification for 5 *B. minax* sample from Pingtang County, Qiannan Buyi and Miao Autonomous Prefecture,

Guizhou Province, Guizhou Province; Lanes 6-10: *B. tsuneonis*-specific primer pair DNA amplification for 5 *B. minax* sample from Pingtang County, Qiannan Buyi and Miao Autonomous Prefecture, Guizhou Province; Lane M: D2000.

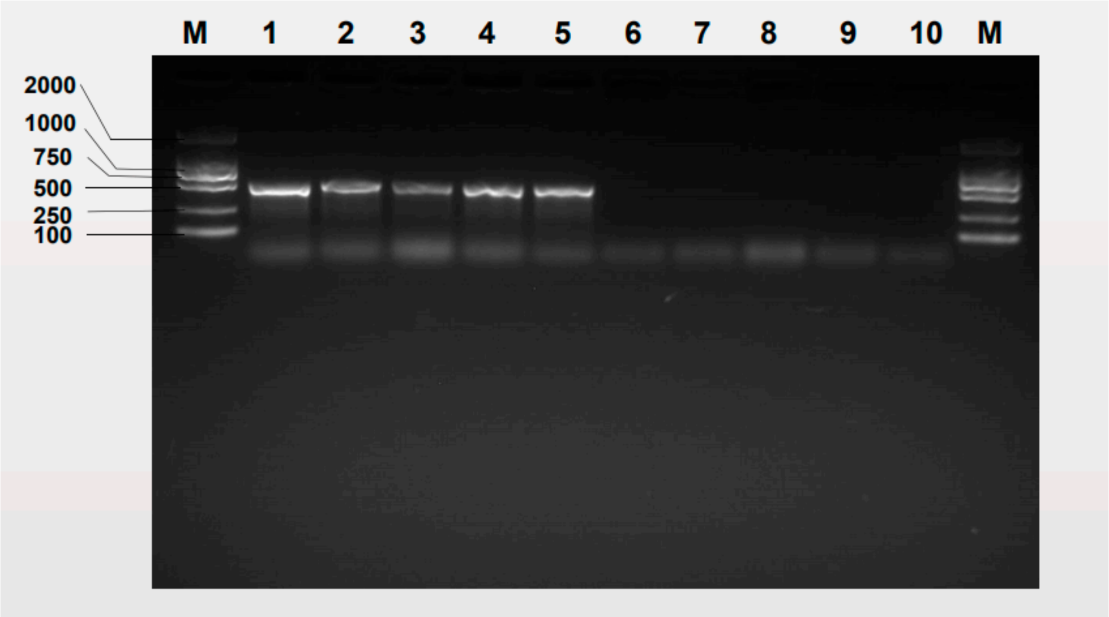

Figure S24 Specificity of the Bm-F/Bm-R *B. minax*-specific primer pair and the Bt-F/Bt-R *B. tsuneonis*-specific primer pair, Lanes 1-5: *B. minax*-specific primer pair DNA amplification for 5 *B. minax* sample from Luodian county, Qiannan Buyi and Miao Autonomous Prefecture, Guizhou Province; Lanes 6-10: *B. tsuneonis*-specific primer pair DNA amplification for 5 *B. minax* sample from Luodian county, Qiannan Buyi and Miao Autonomous Prefecture, Guizhou Province; Lane M: D2000.

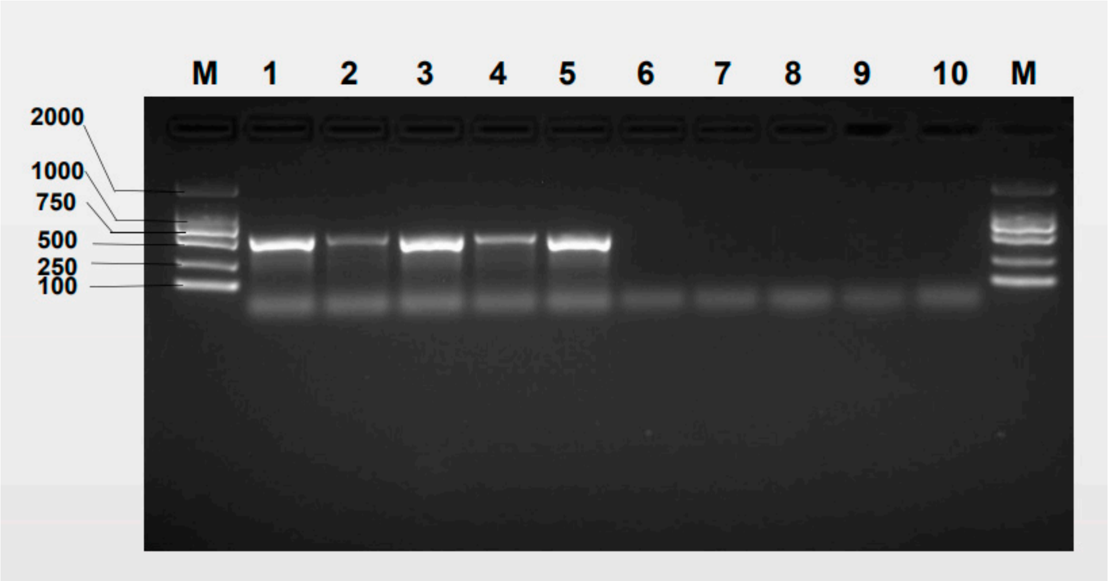

Figure S25 Specificity of the Bm-F/Bm-R *B. minax*-specific primer pair and the Bt-F/Bt-R *B. tsuneonis*-specific primer pair, Lanes 1-5: *B. minax*-specific primer pair DNA amplification for 5 *B. minax* sample from Xifeng County, Guiyang City, Guizhou Province, Guizhou Province; Lanes 6-10: B. tsuneonis-specific primer pair DNA amplification for 5 *B. minax* sample from Xifeng County, Guiyang City, Guizhou Province; Lane M: D2000.

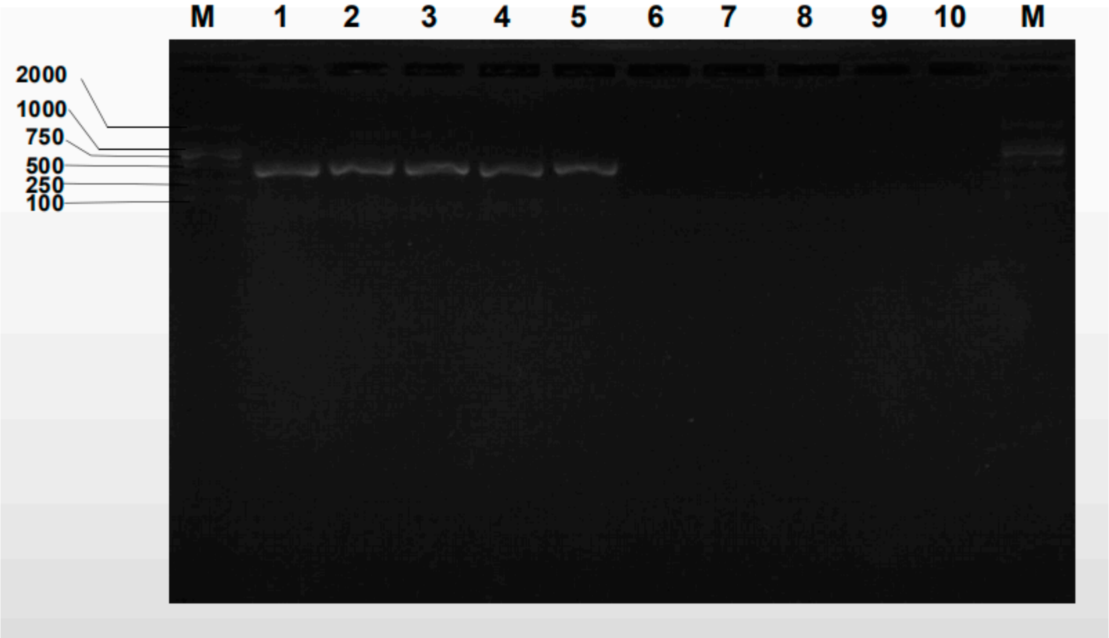

Figure S26 Specificity of the Bm-F/Bm-R *B. minax*-specific primer pair and the Bt-F/Bt-R *B. tsuneonis*-specific primer pair, Lanes 1-5: *B. minax*-specific primer pair DNA amplification for 5 *B. minax* sample from Daguan County, Zhaotong City, Yunnan Province; Lanes 6-10: *B. tsuneonis*-specific primer pair DNA amplification for 5 *B. minax* sample from Daguan County, Zhaotong City, Yunnan Province; Lane M: D2000.

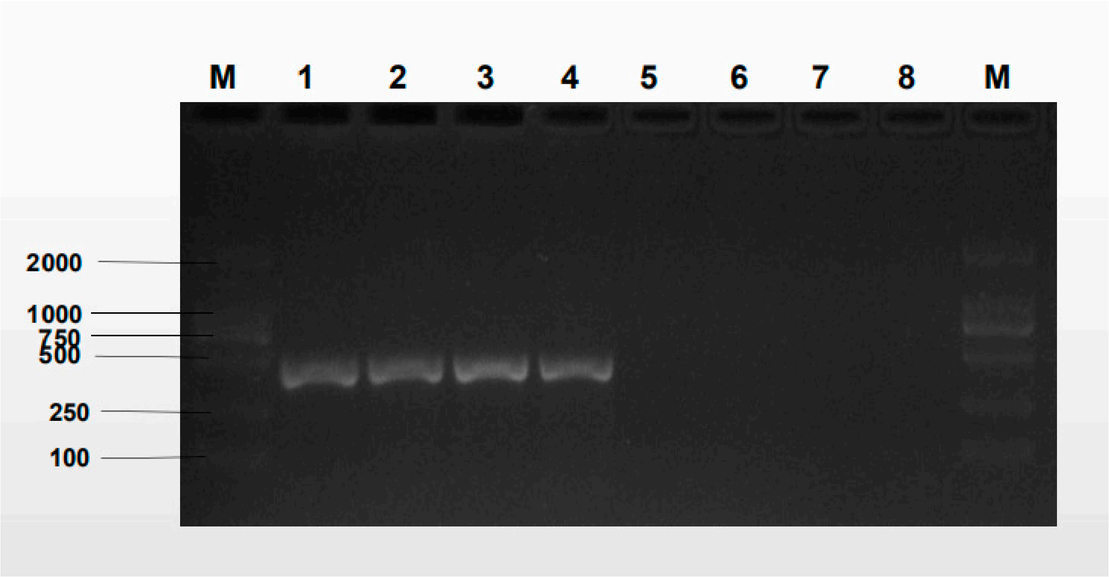

Figure S27 Specificity of the Bm-F/Bm-R *B. minax*-specific primer pair and the Bt-F/Bt-R *B. tsuneonis*-specific primer pair, Lanes 1-4: *B. minax*-specific primer pair DNA amplification for 4 *B. minax* sample from Dianjiang County, Chongqing City; Lanes 5-8: *B. tsuneonis*-specific primer pair DNA amplification for 4 *B. minax* sample from Dianjiang County, Chongqing City; Lane M: D2000.

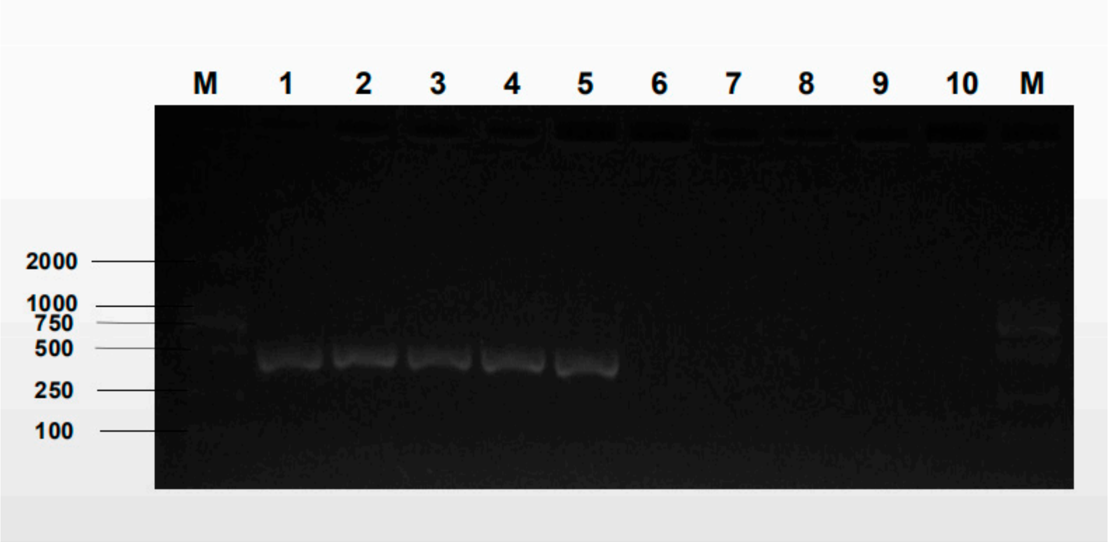

Figure S28 Specificity of the Bm-F/Bm-R *B. minax*-specific primer pair and the Bt-F/Bt-R *B. tsuneonis*-specific primer pair, Lanes 1-5: *B. minax*-specific primer pair DNA amplification for 5 *B. minax* sample from Wanzhou District, Chongqing City; Lanes 6-10: *B. tsuneonis*-specific primer pair DNA amplification for 5 *B. minax* sample from Wanzhou District, Chongqing City; Lane M: D2000.

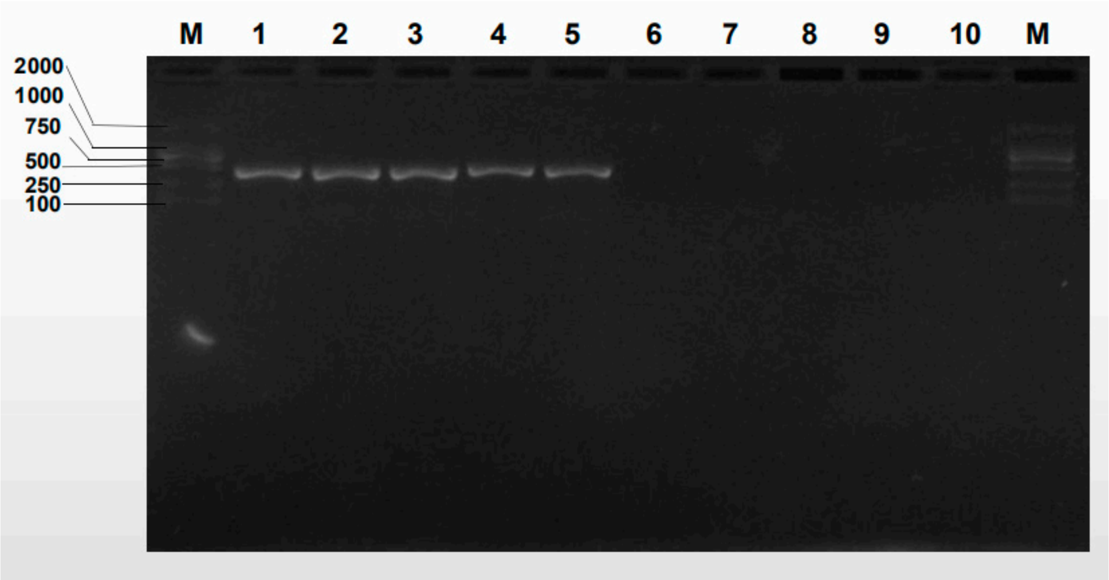

Figure S29 Specificity of the Bm-F/Bm-R *B. minax*-specific primer pair and the Bt-F/Bt-R *B. tsuneonis*-specific primer pair, Lanes 1-5: *B. minax*-specific primer pair DNA amplification for 5 *B. minax* sample from Yiliang County, Zhaotong City, Yunnan Province; Lanes 6-10: *B. tsuneonis*-specific primer pair DNA amplification for 5 *B. minax* sample from Yiliang County, Zhaotong City, Yunnan Province; Lane M: D2000.

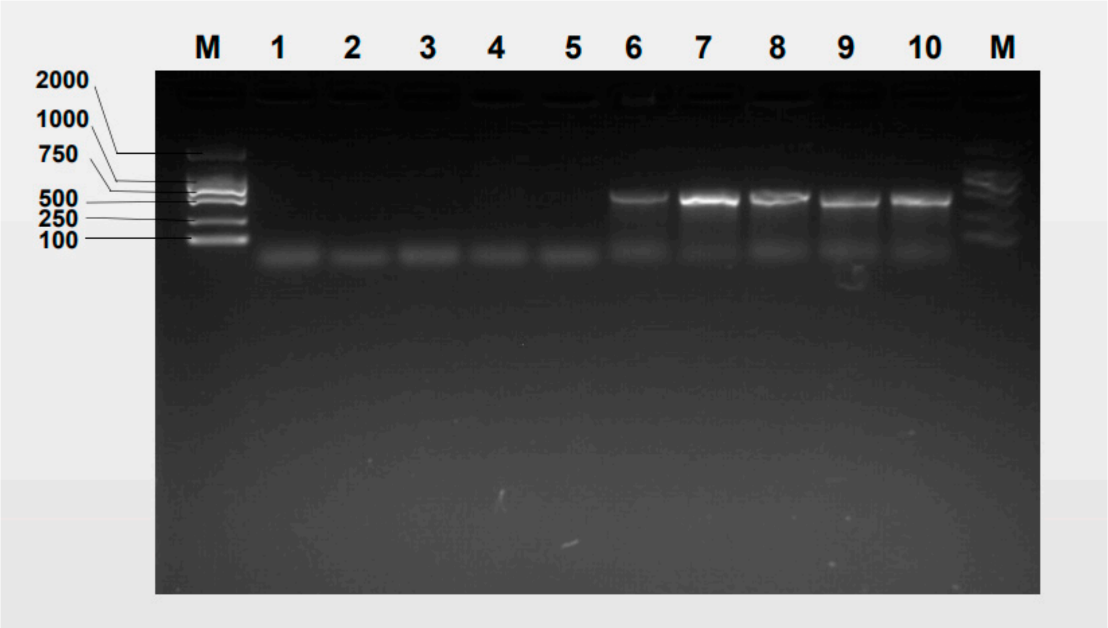

Figure S30 Specificity of the Bm-F/Bm-R *B. minax*-specific primer pair and the Bt-F/Bt-R *B.*

tsuneonis-specific primer pair, Lanes 1-5: *B. minax*-specific primer pair DNA amplification for 5 *B. tsuneonis* sample from Pingshan County, Yibin City, Sichuan Province; Lanes 6-10: *B. tsuneonis*-specific primer pair DNA amplification for 5 *B. tsuneonis* sample from Pingshan County, Yibin City, Sichuan Province; Lane M: D2000.

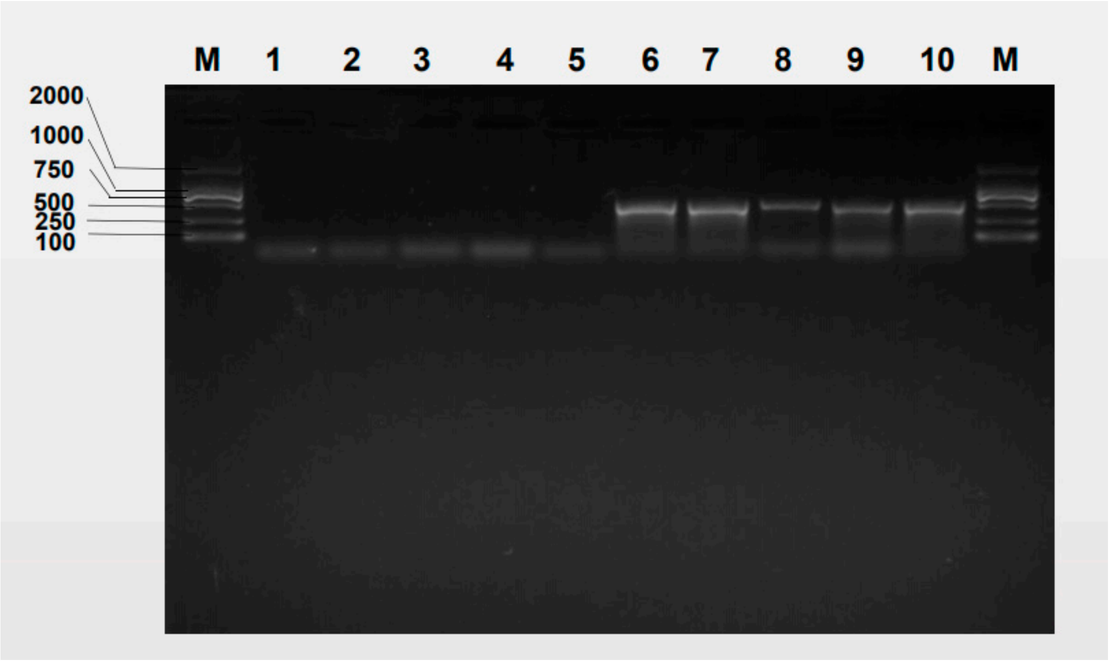

Figure S31 Specificity of the Bm-F/Bm-R *B. minax*-specific primer pair and the Bt-F/Bt-R *B. tsuneonis*-specific primer pair, Lanes 1-5: *B. minax*-specific primer pair DNA amplification for 5 *B. tsuneonis* sample from Libo County, Qiannan Buyi and Miao Autonomous Prefecture, Guizhou Province; Lanes 6-10: *B. tsuneonis*-specific primer pair DNA amplification for 5 *B. tsuneonis* sample from Libo County, Qiannan Buyi and Miao Autonomous Prefecture, Guizhou Province; Lane M: D2000.

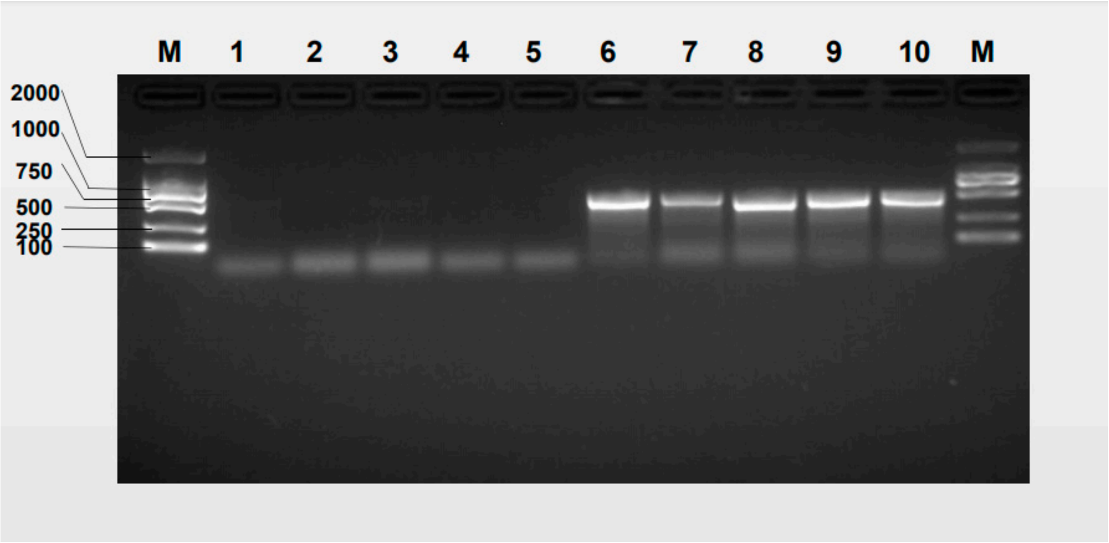

Figure S32 Specificity of the Bm-F/Bm-R *B. minax*-specific primer pair and the Bt-F/Bt-R *B. tsuneonis*-specific primer pair, Lanes 1-5: *B. minax*-specific primer pair DNA amplification for 5 *B. tsuneonis* sample from Jinping County, Qiandongnan Miao and Dong Autonomous Prefecture, Guizhou Province; Lanes 6-10: *B. tsuneonis*-specific primer pair DNA amplification for 5 *B. tsuneonis* sample from Jinping County, Qiandongnan Miao and Dong Autonomous Prefecture, Guizhou Province; Lane M: D2000.

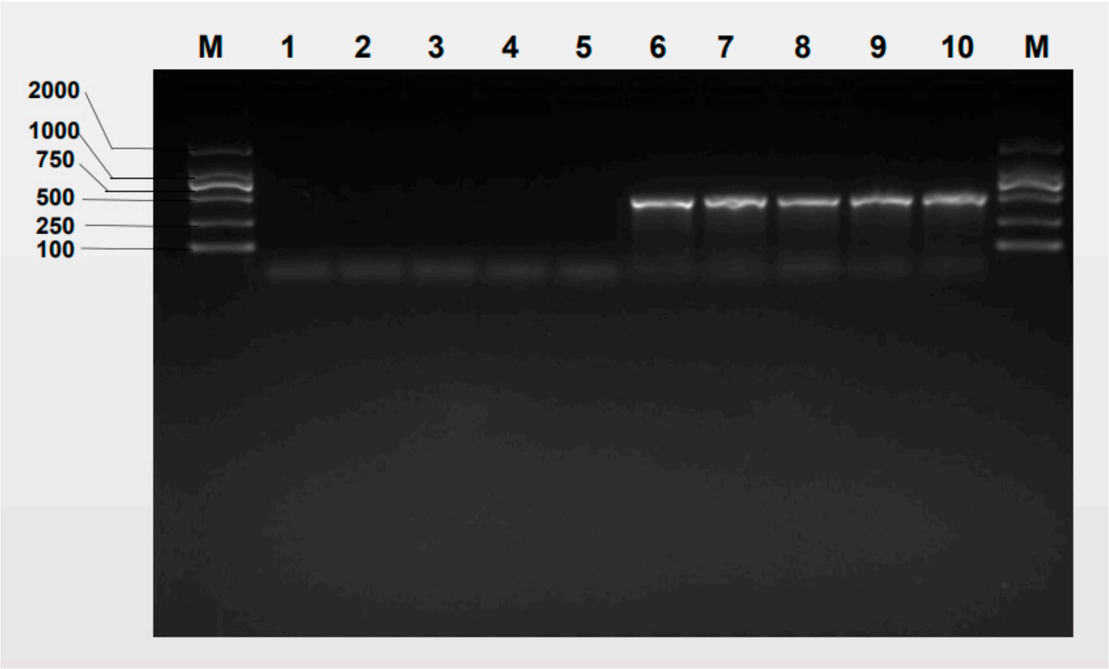

Figure S33 Specificity of the Bm-F/Bm-R *B. minax*-specific primer pair and the Bt-F/Bt-R *B. tsuneonis*-specific primer pair, Lanes 1-5: *B. minax*-specific primer pair DNA amplification for 5 *B. tsuneonis* sample from Yongshan County, Zhaotong City, Yunan Province; Lanes 6-10: *B.*

tsuneonis-specific primer pair DNA amplification for 5 *B. tsuneonis* sample from Yongshan County, Zhaotong City, Yunan Province; Lane M: D2000.

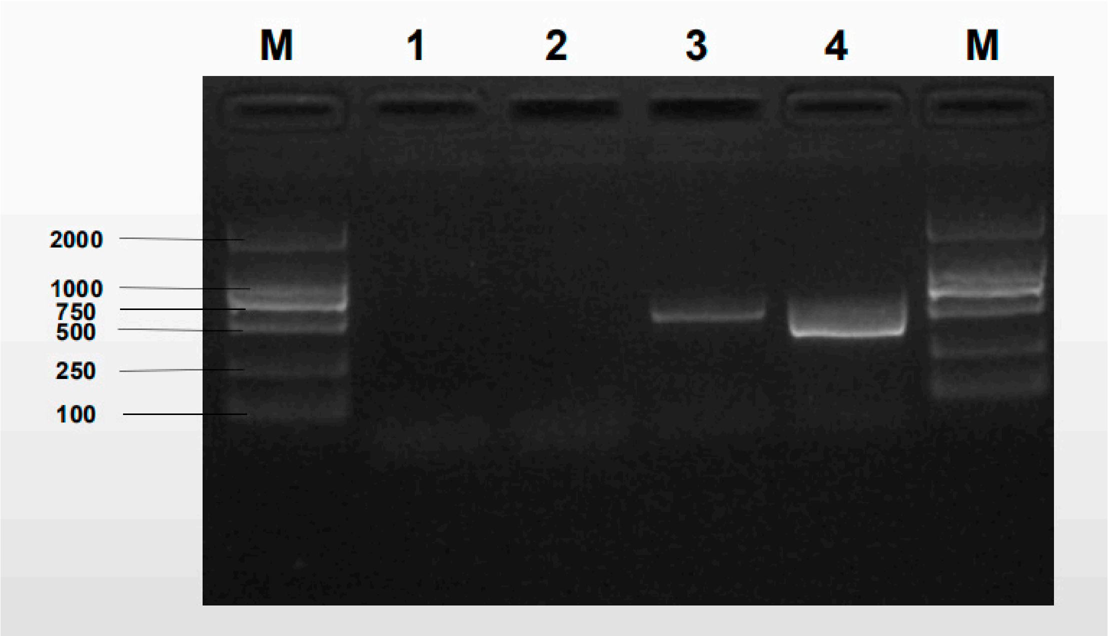

Figure S34 Specificity of the Bm-F/Bm-R *B. minax*-specific primer pair and the Bt-F/Bt-R *B. tsuneonis*-specific primer pair, Lanes 1-2: *B. minax*-specific primer pair DNA amplification for 2 *B. tsuneonis* sample from Chengbu County, Shaoyang City, Hunan Province; Lanes 3-4: *B. tsuneonis*-specific primer pair DNA amplification for 2 *B. tsuneonis* sample from Chengbu County, Shaoyang City, Hunan Province; Lane M: D2000.

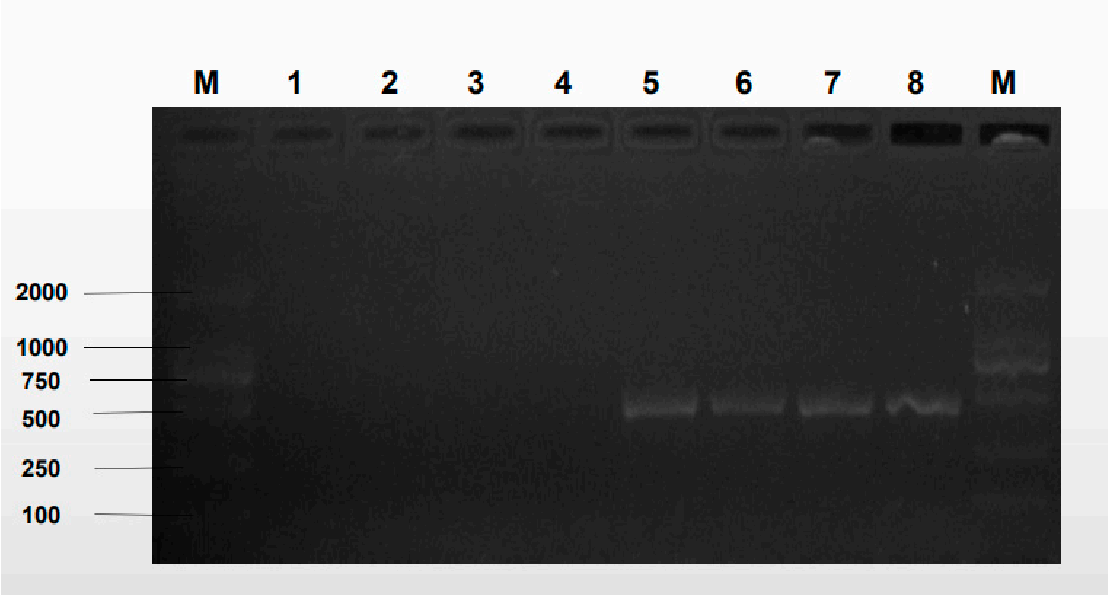

Figure S35 Specificity of the Bm-F/Bm-R *B. minax*-specific primer pair and the Bt-F/Bt-R *B. tsuneonis*-specific primer pair, Lanes 1-4: B. minax-specific primer pair DNA amplification for 4 *B. tsuneonis* sample from Daguan County, Zhaotong City, Yunan Province; Lanes 5-8: *B. tsuneonis*-specific primer pair DNA amplification for 4 *B. tsuneonis* sample from Daguan County, Zhaotong City, Yunan Province; Lane M: D2000.

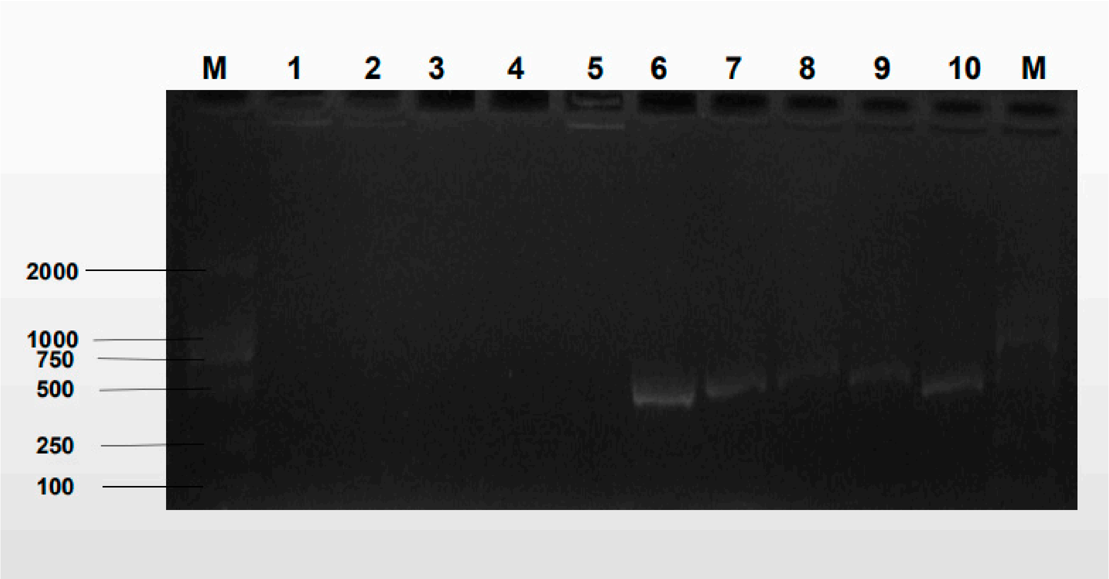

Figure S36 Specificity of the Bm-F/Bm-R *B. minax*-specific primer pair and the Bt-F/Bt-R *B. tsuneonis*-specific primer pair, Lanes 1-5: *B. minax*-specific primer pair DNA amplification for 5 *B. tsuneonis* sample from Yiliang County, Zhaotong City, Yunan Province; Lanes 6-10: *B. tsuneonis*-specific primer pair DNA amplification for 5 *B. tsuneonis* sample from Yiliang County, Zhaotong City, Yunan Province; Lane M: D2000.

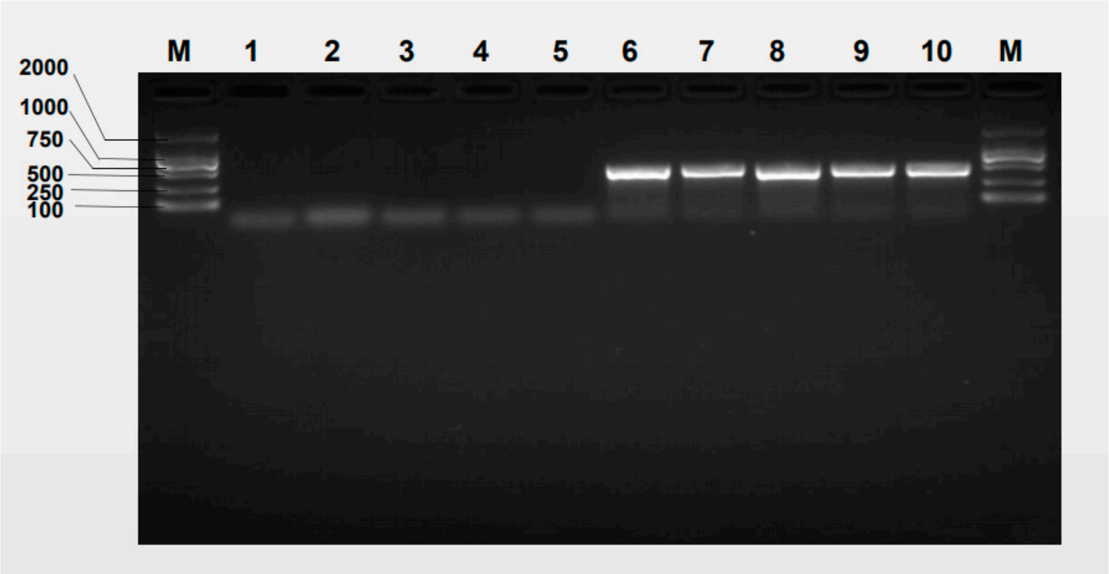

Figure S37 Specificity of the Bm-F/Bm-R *B. minax*-specific primer pair and the Bt-F/Bt-R *B. tsuneonis*-specific primer pair, Lanes 1-5: B. minax-specific primer pair DNA amplification for 5 *B. tsuneonis* sample from Pingxiang County, Chongzuo City, Guangxi Zhuang Autonomous Region; Lanes 6-10: *B. tsuneonis*-specific primer pair DNA amplification for 5 *B. tsuneonis* sample from Pingxiang County, Chongzuo City, Guangxi Zhuang Autonomous Region; Lane M: D2000.

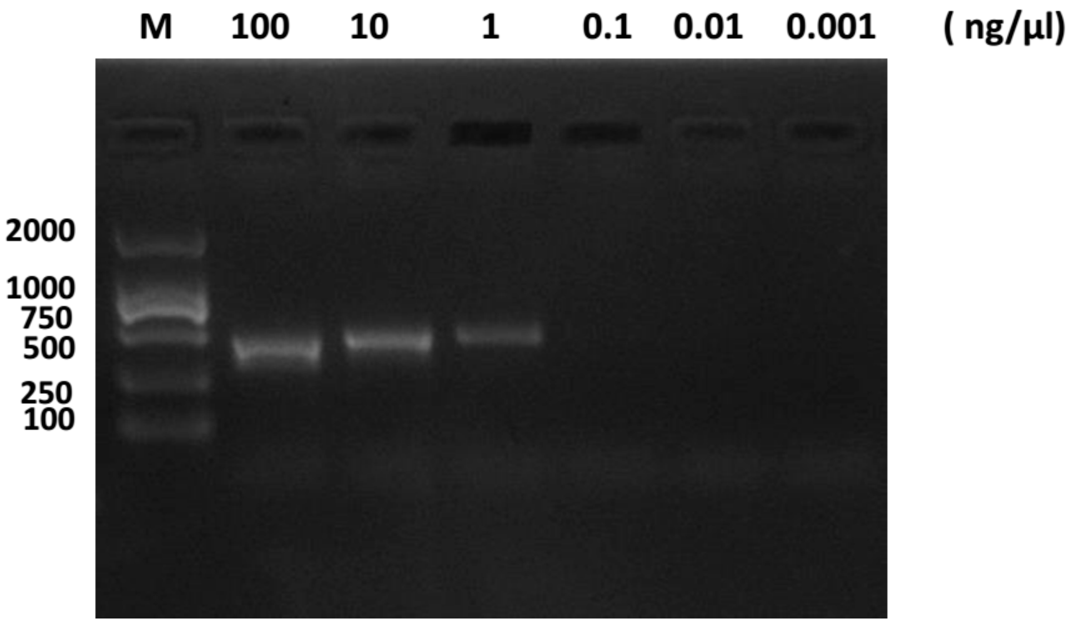

Figure S38 Sensitivity of the Bm-F/ Bm-R *B. minax* species-specific primer pair. The template DNA was from Shaanxi province and its concentrations were as follows: Lane 1: 100 ng/μl ; Lane 2: 10 ng/μl ; Lane 3: 1 ng/μl ; Lane4: 0.1 ng/μl ; Lane 5: 0.01 ng/μl ; Lane 6: 0.001 ng/μl, Lane M: D2000 Marker.

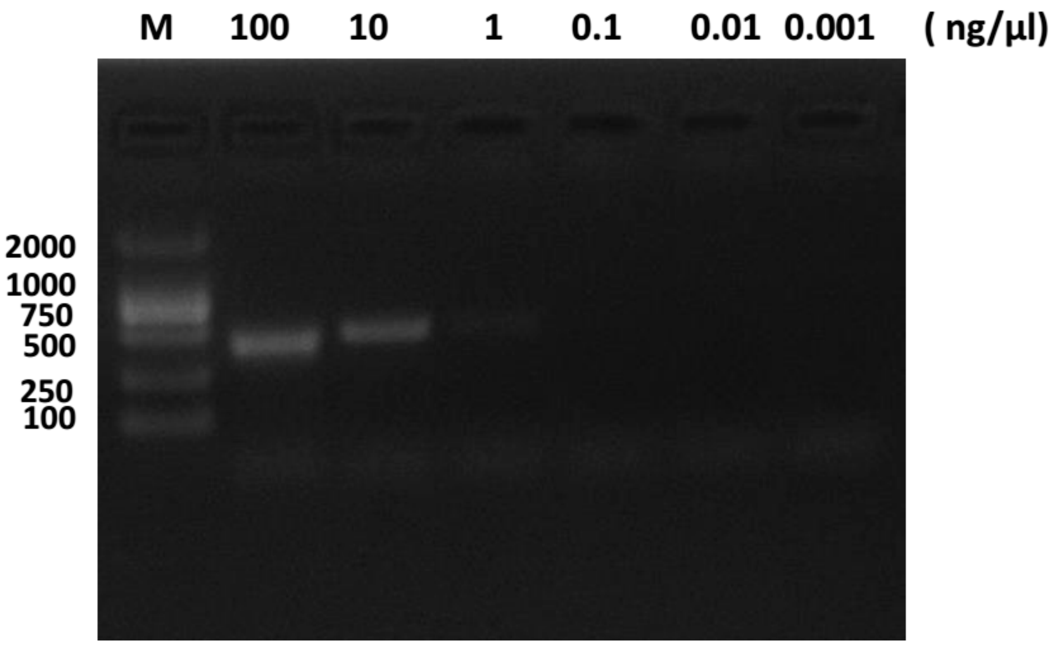

Figure S39 Sensitivity of the Bm-F/ Bm-R *B. minax* species-specific primer pair. The template

DNA was from Hunan Province and its concentrations were as follows: Lane 1: 100 ng/μl ; Lane 2: 10 ng/μl ; Lane 3: 1 ng/μl ; Lane4: 0.1 ng/μl ; Lane 5: 0.01 ng/μl ; Lane 6: 0.001 ng/μl, Lane M: D2000 Marker.

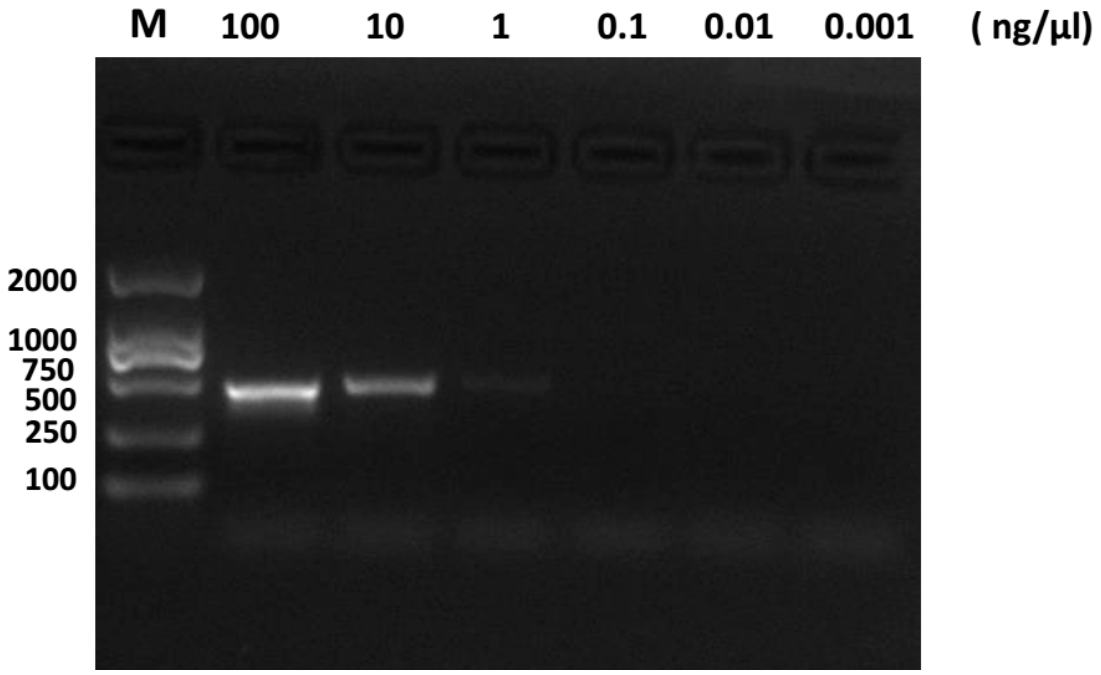

Figure S40 Sensitivity of the Bm-F/ Bm-R *B. minax* species-specific primer pair. The template DNA was from Guizhou Province and its concentrations were as follows: Lane 1: 100 ng/μl ; Lane 2: 10 ng/μl ; Lane 3: 1 ng/μl ; Lane4: 0.1 ng/μl ; Lane 5: 0.01 ng/μl ; Lane 6: 0.001 ng/μl, Lane M: D2000 Marker.

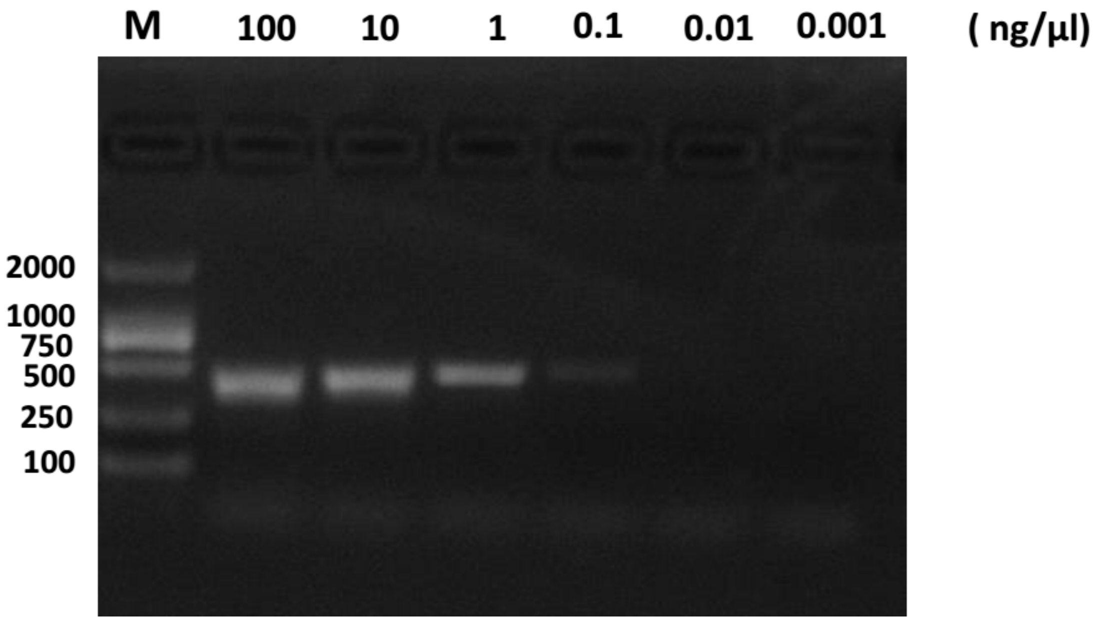

Figure S41 Sensitivity of the Bm-F/ Bm-R *B. minax* species-specific primer pair. The template DNA was from Chongqing City and its concentrations were as follows: Lane 1: 100 ng/μl ; Lane 2: 10 ng/μl ; Lane 3: 1 ng/μl ; Lane4: 0.1 ng/μl ; Lane 5: 0.01 ng/μl ; Lane 6: 0.001 ng/μl, Lane M: D2000 Marker.

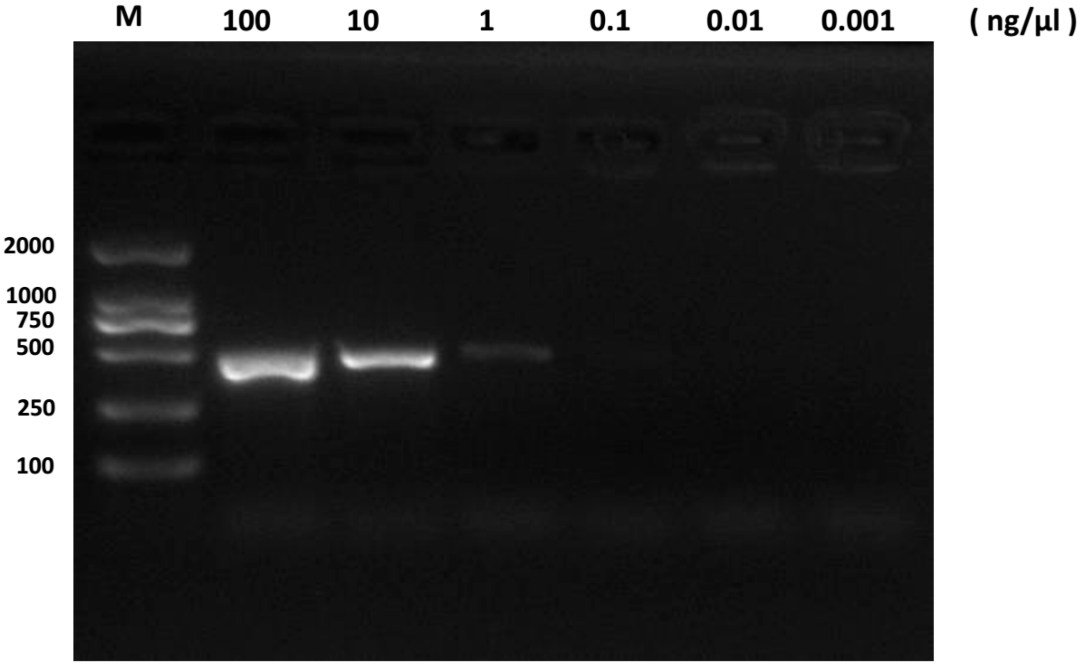

Figure S42 Sensitivity of the Bm-F/ Bm-R *B. minax* species-specific primer pair. The template DNA was from Yunnan Province and its concentrations were as follows: Lane 1: 100 ng/μl ; Lane 2: 10 ng/μl ; Lane 3: 1 ng/μl ; Lane4: 0.1 ng/μl ; Lane 5: 0.01 ng/μl ; Lane 6: 0.001 ng/μl, Lane M:

D2000 Marker.

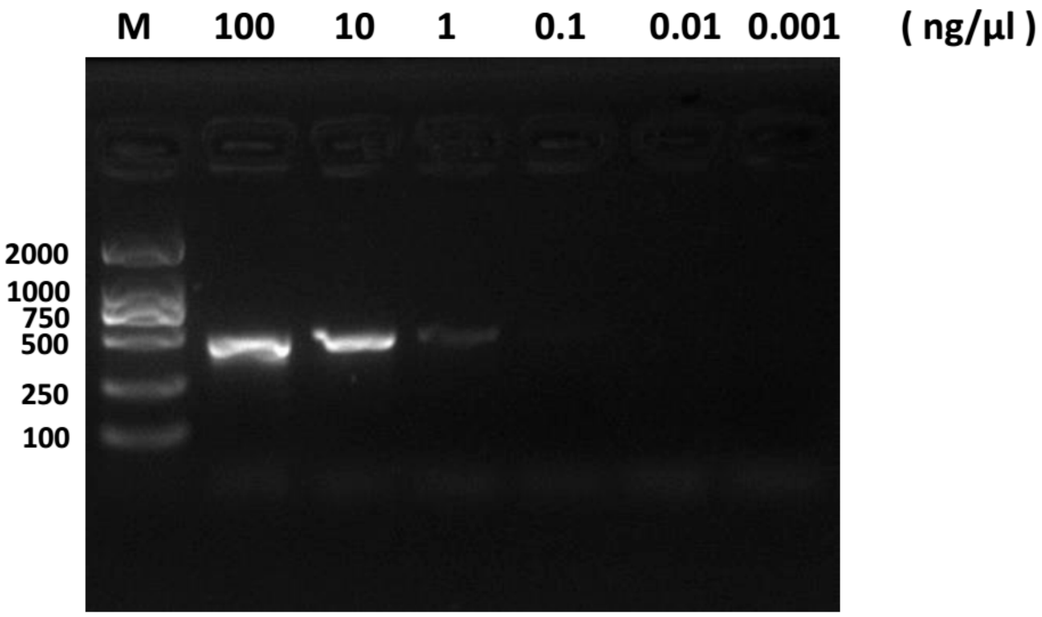

Figure S43 Sensitivity of the Bm-F/ Bm-R *B. minax* species-specific primer pair. The template DNA was from Hubei Province and its concentrations were as follows: Lane 1: 100 ng/μl ; Lane 2: 10 ng/μl ; Lane 3: 1 ng/μl ; Lane4: 0.1 ng/μl ; Lane 5: 0.01 ng/μl ; Lane 6: 0.001 ng/μl, Lane M: D2000 Marker.

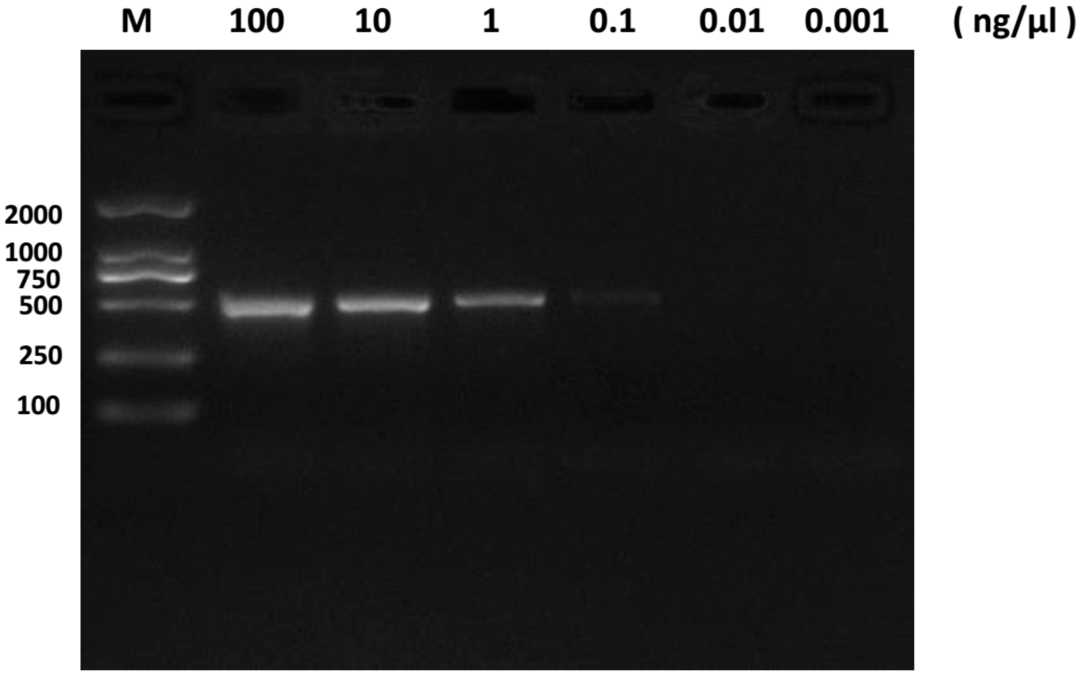

Figure S44 Sensitivity of the Bt-F/ Bt-R *B. tsuneonis* species-specific primer pair. The template DNA was from Sichuan Province and its concentrations were as follows: Lane 1: 100 ng/μl ; Lane 2: 10 ng/μl ; Lane 3: 1 ng/μl ; Lane4: 0.1 ng/μl ; Lane 5: 0.01 ng/μl ; Lane 6: 0.001 ng/μl, Lane M: D2000 Marker.

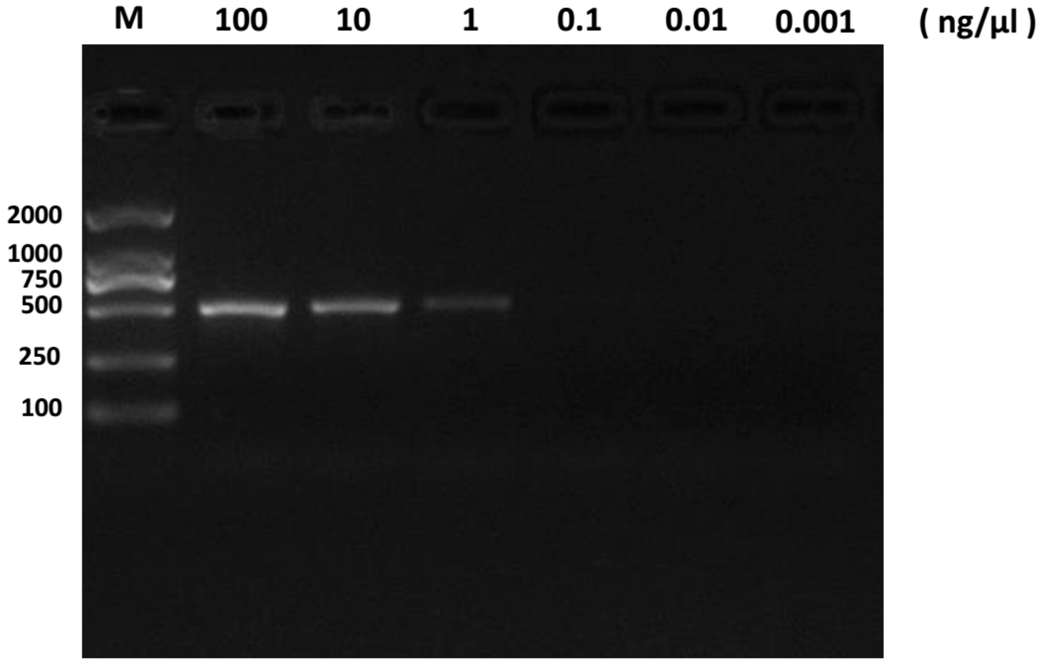

Figure S45 Sensitivity of the Bt-F/ Bt-R *B. tsuneonis* species-specific primer pair. The template DNA was from Yunnan Province and its concentrations were as follows: Lane 1: 100 ng/μl ; Lane

2: 10 ng/μl ; Lane 3: 1 ng/μl ; Lane4: 0.1 ng/μl ; Lane 5: 0.01 ng/μl ; Lane 6: 0.001 ng/μl, Lane M: D2000 Marker.

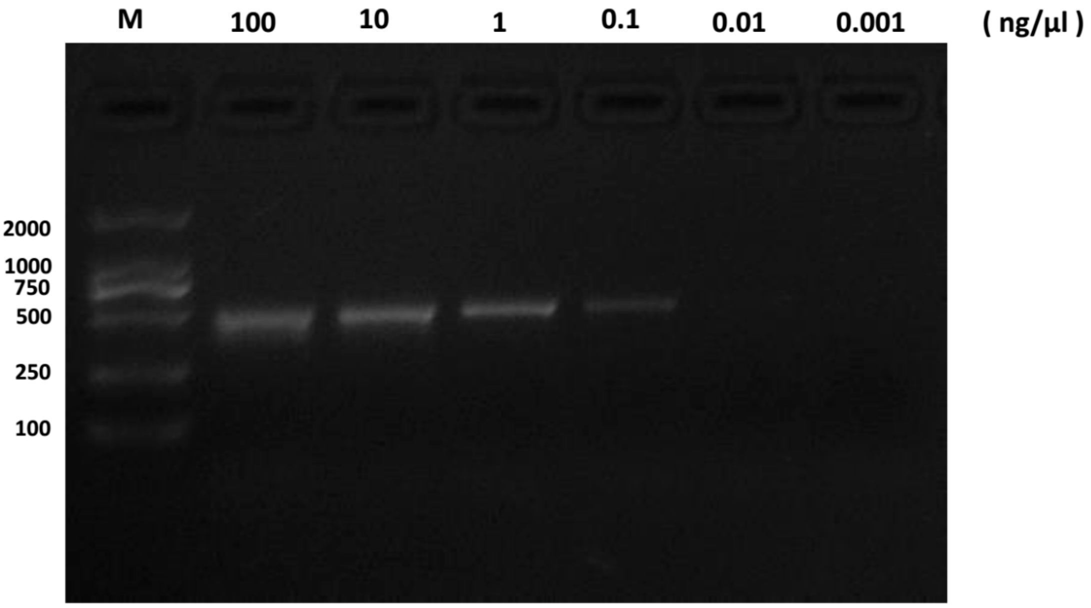

Figure S46 Sensitivity of the Bt-F/ Bt-R *B. tsuneonis* species-specific primer pair. The template DNA was from Hunan Province and its concentrations were as follows: Lane 1: 100 ng/μl ; Lane 2: 10 ng/μl ; Lane 3: 1 ng/μl ; Lane4: 0.1 ng/μl ; Lane 5: 0.01 ng/μl ; Lane 6: 0.001 ng/μl, Lane M: D2000 Marker.

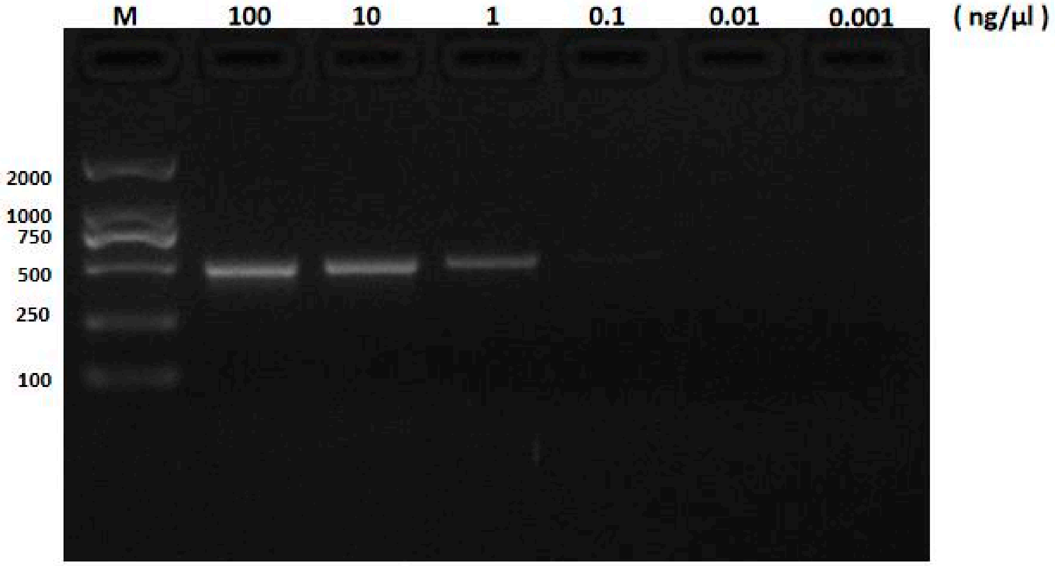

Figure S47 Sensitivity of the Bt-F/ Bt-R *B. tsuneonis* species-specific primer pair. The template DNA was from Guangxi Zhuang Autonomous Region and its concentrations were as follows: Lane 1: 100 ng/μl ; Lane 2: 10 ng/μl ; Lane 3: 1 ng/μl ; Lane4: 0.1 ng/μl ; Lane 5: 0.01 ng/μl ; Lane 6: 0.001 ng/μl, Lane M: D2000 Marker.

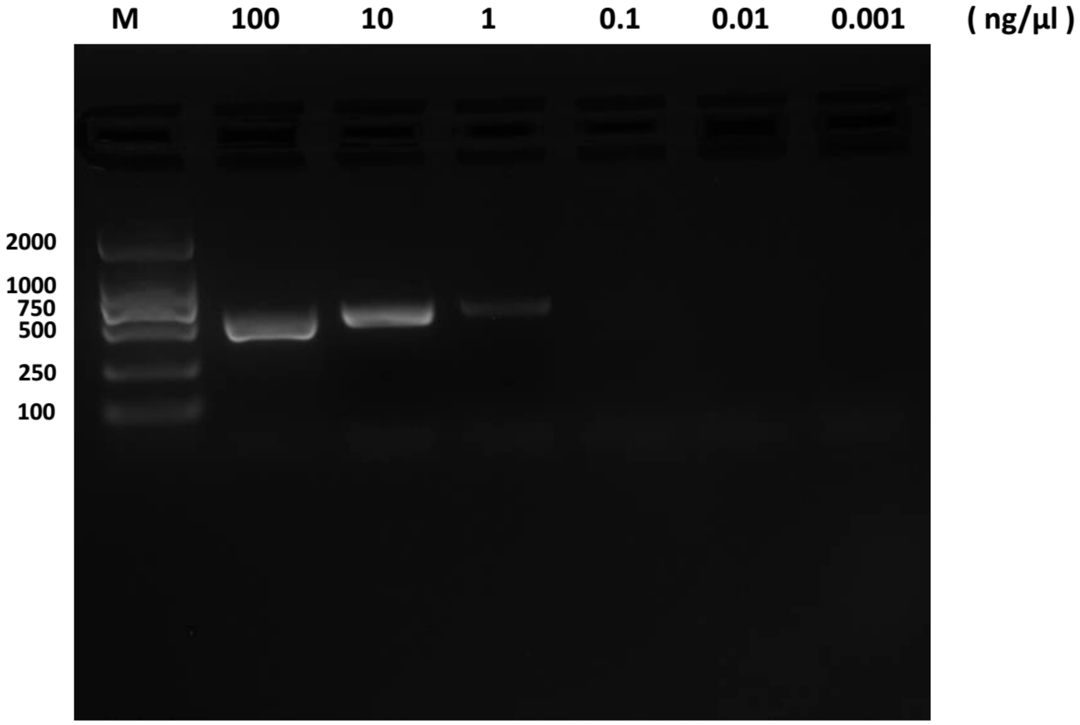

Figure S48 Sensitivity of the Bt-F/ Bt-R *B. tsuneonis* species-specific primer pair. The template

DNA was from Guizhou Province and its concentrations were as follows: Lane 1: 100 ng/μl ; Lane 2: 10 ng/μl ; Lane 3: 1 ng/μl ; Lane4: 0.1 ng/μl ; Lane 5: 0.01 ng/μl ; Lane 6: 0.001 ng/μl, Lane M: D2000 Marker.
